# New species of *Agathodesmus* Silvestri, 1910 from Australia (Diplopoda, Polydesmida, Haplodesmidae)

**DOI:** 10.3897/zookeys.325.5932

**Published:** 2013-08-20

**Authors:** Robert Mesibov

**Affiliations:** 1 Queen Victoria Museum and Art Gallery, Launceston, Tasmania, Australia 7250

**Keywords:** Millipede, Diplopoda, Polydesmida, Haplodesmidae, Australia

## Abstract

The genus *Agathodesmus* Silvestri, 1910 is speciose and widespread in high-rainfall parts of eastern Australia. In addition to the type species *Agathodesmus steeli* Silvestri, 1910 and *Agathodesmus johnsi* Mesibov, 2009 from New South Wales and *Agathodesmus bucculentus* (Jeekel, 1986) from Queensland, the following 18 new species are recognised: *Agathodesmus adelphus* **sp. n.**, *Agathodesmus aenigmaticus* **sp. n.**, *Agathodesmus agnus* **sp. n.**, *Agathodesmus anici* **sp. n.**, *Agathodesmus gayundah* **sp. n.**, *Agathodesmus hahnensis* **sp. n.**, *Agathodesmus kerensis* **sp. n.**, *Agathodesmus kirrama* **sp. n.**, *Agathodesmus millaa* **sp. n.**, *Agathodesmus parapholeus* **sp. n.**, *Agathodesmus quintanus* **sp. n.**, *Agathodesmus sagma* **sp. n.**, *Agathodesmus summus* **sp. n.** and *Agathodesmus yuccabinensis* **sp. n.** from Queensland; *Agathodesmus carorum* **sp. n.** from New South Wales and Victoria; *Agathodesmus bonang* **sp. n.** and *Agathodesmus morwellensis* **sp. n.** from Victoria; and *Agathodesmus chandleri* **sp. n.** from South Australia.

## Introduction

*Agathodesmus* Silvestri, 1910 was established for a single female of *Agathodesmus steeli* Silvestri, 1910, a small polydesmidan millipede from New South Wales, Australia. In a recent paper (Mesibov 2009) I redescribed *Agathodesmus steeli* from freshly collected males and females, and added a second New South Wales species to the genus. The two species share with *Atopogonus baccatus* Carl, 1926 (New Caledonia) and *Agathodesmus bucculentus* Jeekel, 1986 (Queensland, Australia) a distinctive gonopod conformation: there is no cannula or prostatic groove, and the telopodite is sharply bent mid-length at a 'knee' topped with a thin tab. In Mesibov (2009) I made *Atopogonus* Carl, 1926 a junior synonym of *Agathodesmus*.

In this paper I describe 18 new species of *Agathodesmus* and add a few observations on *Agathodesmus bucculentus*. I collected specimens of four of the new species during field trips from 2006 to 2011. The remaining 14 species were found in the Australian National Insect Collection and the Queensland Museum, mainly among arthropods extracted from rainforest litter by the Berlese method.

**Figure 1.**
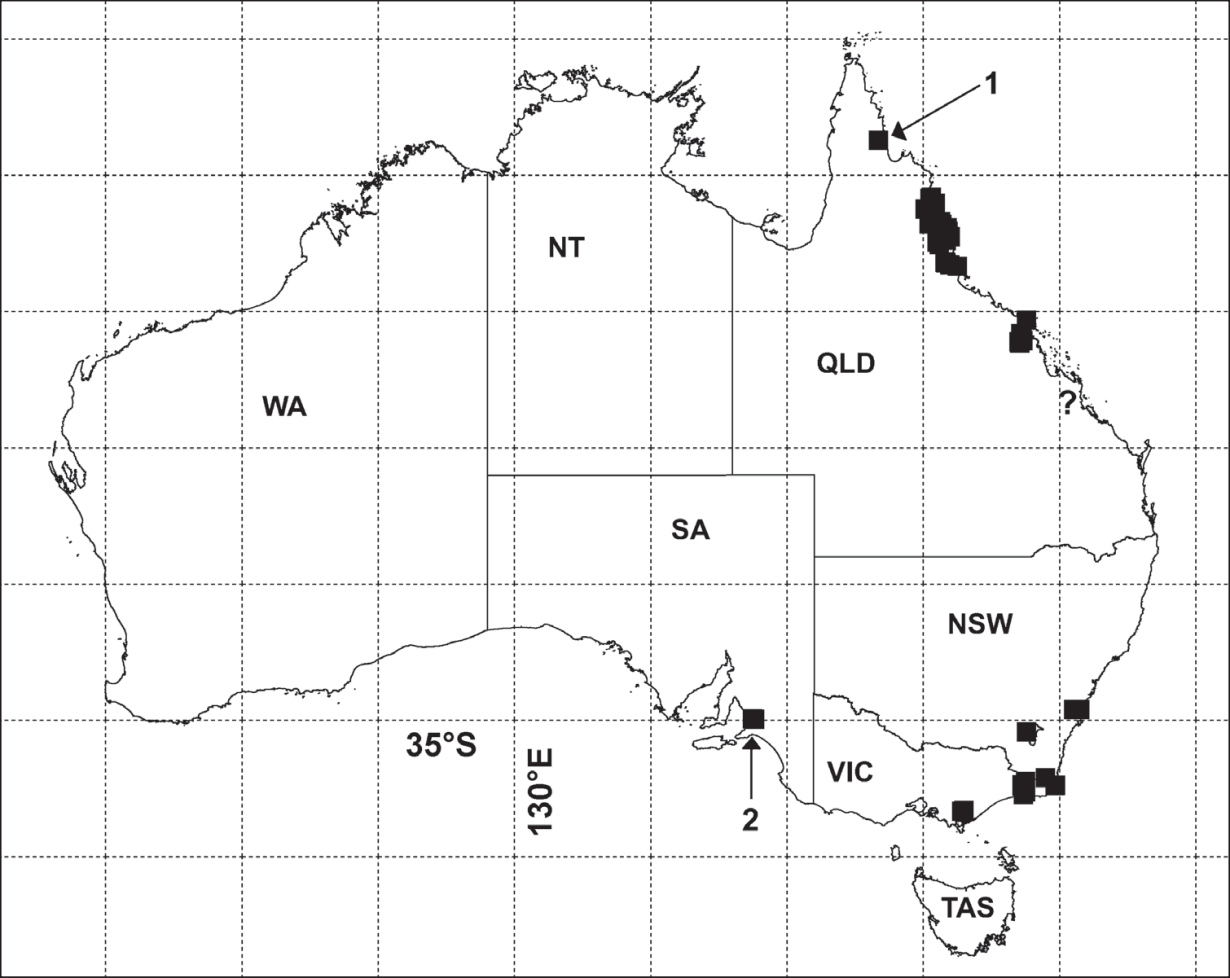
Localities for *Agathodesmus* spp. in Australia (filled black squares) as of July 2012. **1** = only known locality for *Agathodesmus anici* sp. n. **2** = cluster of 4 localities for *Agathodesmus chandleri* sp. n., **?** = questionable Cammoo Caves locality for *Agathodesmus agnus* sp. n.; see Figs 11-13 for other species. Geographic projection, 5° latitude-longitude grid. NSW = New South Wales, NT = Northern Territory, QLD = Queensland, SA = South Australia, TAS = Tasmania, VIC = Victoria, WA = Western Australia.

The late C.A.W. Jeekel wrote of *Agathodesmus bucculentus*: “With the discovery of a species of *Atopogonus* in what seems to be a perfectly natural habitat in Queensland it becomes likely that the genus is in essence a continental Australian taxon” (Jeekel 1986, p. 46). *Agathodesmus* is now known to be widespread and speciose in parts of eastern Australia with a mean annual rainfall greater than 1000 mm (Fig. 1). *Agathodesmus* is particularly diverse in the Wet Tropics of far north Queensland, which is home to 12 of the 21 known Australian species. The wide gaps in the genus distribution map suggest to me that more species remain to be discovered, especially in the wetter mountain forests of New South Wales, southeast Queensland and Victoria.

## Methods

'Male' and 'female' in the text refer to adult individuals. All specimens are stored in 75–80% ethanol in their respective repositories.

Gonopods were cleared in 80% lactic acid and temporarily mounted in 60% lactic acid for optical microscopy, while other body parts were temporarily mounted in a 1:1 glycerol:water mixture. Preliminary gonopod drawings were traced from photomicrographs taken at 160× through a binocular microscope. Measurements were made with a Nikon SMZ800 binocular dissecting microscope using an eyepiece scale, and are reported below to the nearest 0.5 mm. SEM images were acquired digitally using a FEI Quanta 600; some specimens were examined after air-drying and sputter-coating with platinum, while others were air-dried, examined and returned to alcohol. Images and drawings were prepared for publication using GIMP 2.8. Maps were generated using ArcView GIS 3.2.

The Appendix tabulates specimen data for all museum lots noted in the text. Locality details are given in all cases with latitude and longitude based on the WGS84 datum. Latitude/longitude data for Queensland Museum specimen localities are mainly taken from the Queensland Museum insect collection database, but some Mt Bellenden Ker data have been corrected following Mesibov (2012b). My estimate of the uncertainty for each locality is the radius of a circle around the stated position, in metres or kilometres.

Abbreviations: AM = Australian Museum, Sydney, NSW; ANIC = Australian National Insect Collection, Canberra, Australian Capital Territory; ANZSES = Australia New Zealand Scientific Exploration Society; NMV = Museum Victoria, Melbourne, Vic; NSW = New South Wales; Qld = Queensland; QM = Queensland Museum, Brisbane, Qld; SA = South Australia; SAM = South Australian Museum, Adelaide, SA; Vic = Victoria.

## Taxonomy

### Order Polydesmida Pocock, 1887
Suborder Polydesmidea Pocock, 1887
Family Haplodesmidae Cook, 1895

#### Agathodesmus

Silvestri, 1910

http://species-id.net/wiki/Agathodesmus

Agathodesmus : Silvestri 1910:362. Attems 1914:282, 1940:487. Brölemann 1916:547, 587. Jeekel 1971:310; 1982:11; 1983:146; 1985:50, 51; 1986:46. Hoffman 1980:184. Simonsen 1990:57. Golovatch et al. 2009:2. Mesibov 2009:92.
Atopogonus : Carl 1926:386. Attems 1940:477. Verhoeff 1941:406. Jeekel 1971:314, 1984:88, 1986:46. Hoffman 1980:186, 1999:480. Simonsen 1990:57. Golovatch et al. 2001:185, 2009:2, 44. Mesibov 2009:92 (synonymised).

##### Type species.

*Agathodesmus steeli* Silvestri, 1910, by original designation; of *Atopogonus*, *Atopogonus baccatus* (Carl, 1926), by monotypy.

Other assigned species: *Agathodesmus adelphus* sp. n., *Agathodesmus aenigmaticus* sp. n., *Agathodesmus agnus* sp. n., *Agathodesmus anici* sp. n., *Agathodesmus bonang* sp. n., *Agathodesmus bucculentus* (Jeekel, 1986), *Agathodesmus carorum* sp. n., *Agathodesmus chandleri* sp. n., *Agathodesmus gayundah* sp. n., *Agathodesmus hahnensis* sp. n., *Agathodesmus johnsi* Mesibov, 2009, *Agathodesmus kerensis* sp. n., *Agathodesmus kirrama* sp. n., *Agathodesmus millaa* sp. n., *Agathodesmus morwellensis* sp. n., *Agathodesmus parapholeus* sp. n., *Agathodesmus quintanus* sp. n., *Agathodesmus sagma* sp. n., *Agathodesmus summus* sp. n., *Agathodesmus yuccabinensis* sp. n.

##### Diagnosis.

Small Polydesmida with head and 19 or 20 rings; body not curling in spiral; head and telson facing downwards; metatergites with numerous tubercles of varying sizes, sometimes bearing a single seta; ring 2 tergite extended laterally, basally and anteriorly, and edged with large tubercles; no paranota on posterior rings, but 'pseudo-paranota' of metatergal tubercles sometimes present above leg bases; gonopod with neither cannula nor prostatic groove, telopodites separate, each consisting of a more or less cylindrical proximal portion tipped with a thin tab, and a lamellar distal portion arising near the apex of the proximal portion and directed posterobasally or laterobasally.

##### Remarks.

**Figure 2.**
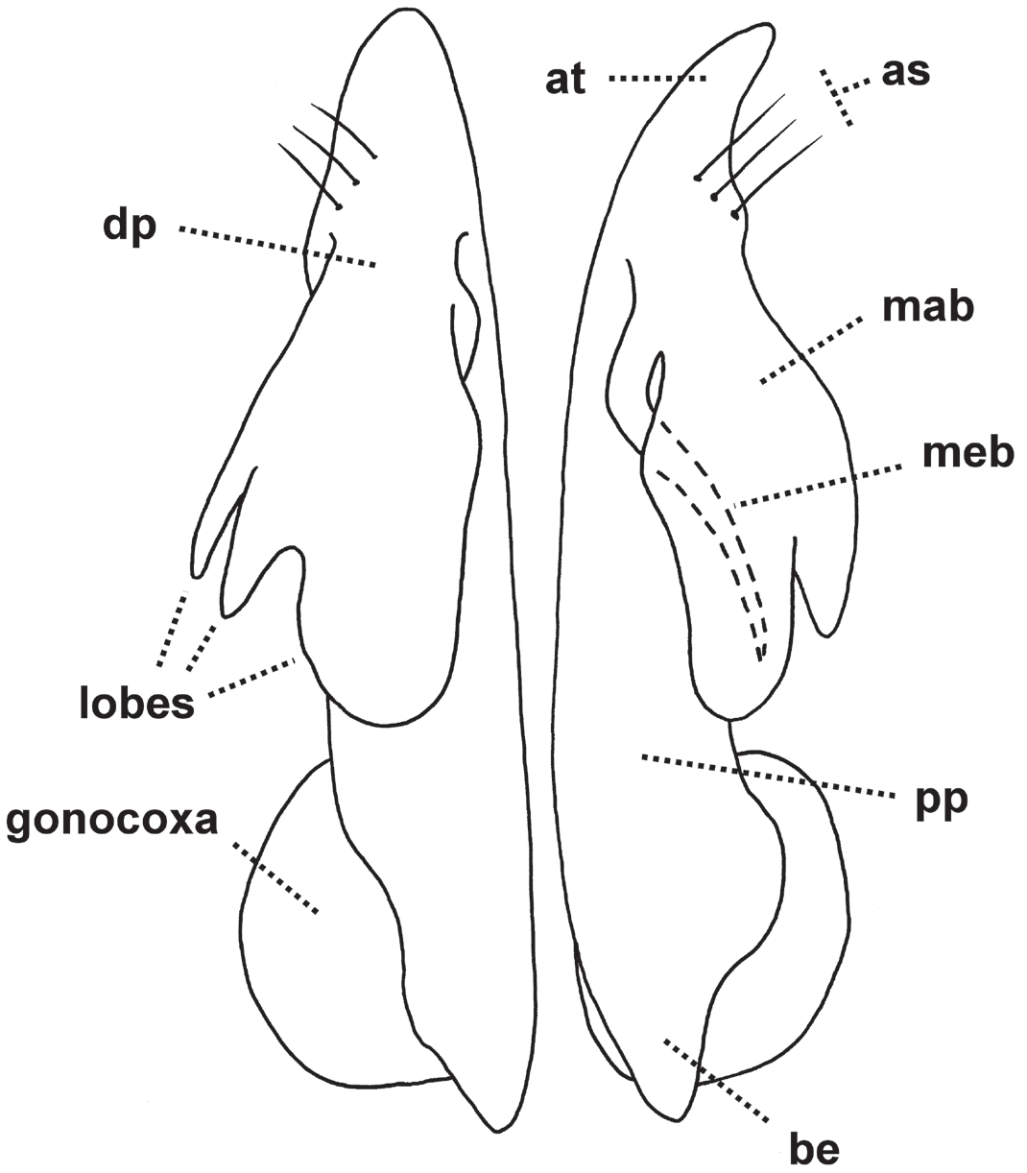
Generalised gonopods of *Agathodesmus* spp., posterior view. **as** = apical setae, **at** = apical tab, **be** = basal extension of telopodite, **dp** = distal portion of telopodite, **mab** = main branch of distal portion of telopodite, **meb** = median branch of distal portion of telopodite, **pp** = proximal portion of telopodite.

The diagnosis above slightly amends the one given in Mesibov (2009). *Agathodesmus* as a genus is easily recognised by the distinctive structure of the gonopod telopodite (Fig. 2). The proximal portion (**pp**) is typically straight and usually more or less cylindrical, but with the medial and posterior surfaces flattened. The **pp** arises from the distomedial corner of the small, oblong gonocoxa, where its base is partly contained in a small concavity. The telopodite base may extend basally as a short, rounded projection (**be**) to overlap the apex of the gonocoxa in ventral or posterior view. The apex of the **pp** extends distally as a thin tab (**at**), and on or just below the tab on the posterior surface there are three long, closely adjacent, apical setae (**as**) in a row; scattered smaller setae may be present on the posterior surface of the **pp**. Arising just basal to the **as** on the posterodistal surface of the **pp** is the distal portion (**dp**) of the telopodite. Jeekel (1986) used the word 'complicated' three times in his description of the **dp** ('acropodite') in *Agathodesmus bucculentus*, and while the details of its structure can be very hard to put into words, some generalities are clear and are applicable to all known Australian *Agathodesmus* species. The **dp** is always directed posterobasally or laterobasally, giving the telopodite as a whole the appearance of a leg tightly bent at the knee. The main branch (**mab**) of the **dp** is flattened into a lamella and is usually divided into lobes. The lamella is typically curved so that the surface seen in posterior view is slightly convex, and the distal margin of the **mab** and portions of its inner, concave surface may be thickened or folded. A smaller, medial branch (**meb)** of the **dp** arises near the base of the **dp** on its medial side and usually curves laterally so that its tip is hidden behind the **mab** in ventral view. Portions of the **meb** are sometimes densely covered with fine, hair-like structures.

In this paper I provide posterior or posterolateral gonopod views of all species described here. These views are convenient when examining males with tightly flexed telopodites, and with careful attention to shape and position, a posterior or posterolateral view is diagnostic. However, readers should be aware that important diagnostic details of the telopodites may be hidden in these views, and that in the SEM views published here, thin portions of the **mab** may have been distorted by drying. *Agathodesmus* spp. are unusual among Australian Polydesmida in their 'hidden' gonopod complexity, and manipulation of the telopodite to partially unbend it and separate the **meb** is often necessary for a positive identification.

When an *Agathodesmus* sp. gonopod is cleared with 80% lactic acid, the telopodite sometimes extends so that the angle between the **pp** and **dp** is greater than 90°. It is easy to imagine that the telopodite extends in this way during mating, and that the apical tab (**at**) serves as a check on the rotation of the telopodite 'knee'. A tab in a similar location is present anterior to the 'joint' in the pseudo-articulated gonopod telopodite of species in the Australian genus *Ginglymodesmus* (Dalodesmidae) (Mesibov 2005). However, I have as yet no evidence to support the idea that the telopodite extends in living males. No *Agathodesmus* have been collected and preserved in copula, and in all of the 430+ *Agathodesmus* males I have examined the uncleared gonopod telopodites are flexed.

Variation in most non-gonopod characters across the genus is minor and the redescription of the type species *Agathodesmus steeli* in Mesibov (2009) applies to most details in the new species. The 'diagnostic descriptions' given below include only those characters known to vary significantly among Australian *Agathodesmus* spp.

In 12 of the 21 known Australian species, males and females have 19 body rings, in five species 20 rings, and in one species males have 19 rings and females 20; three species known only from males have 19, 19 and 20 rings. The smaller species generally have 19 rings and the larger 20 rings, but the correlation of ring number and body size is loose, and two of the largest Australian species have 19 rings (*Agathodesmus kirrama* sp. n. and *Agathodesmus yuccabinensis* sp. n.).

**Figure 3.**
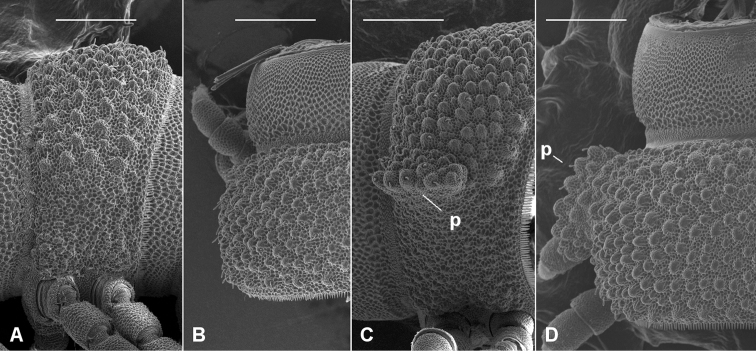
'Pseudo-paranotum' extremes. Lateral (**A**, **C**) and dorsal (**B**, **D**) views of midbody rings of *Agathodesmus bucculentus* (Jeekel, 1986) (**A**, **B**; no pseudo-paranotum; male ex ANIC 64-000332) and *Agathodesmus gayundah* sp. n. (**C**, **D**; wide pseudo-paranotum, **p**; male paratype ex QM S96038). Scale bars = 0.25 mm; **A** reversed (left-right) for clarity.

There is also no apparent relationship between body size and the development (or absence) of the 'pseudo-paranota' formed by lateral metatergal tubercles on posterior rings (Fig. 3).

**Figure 4.**
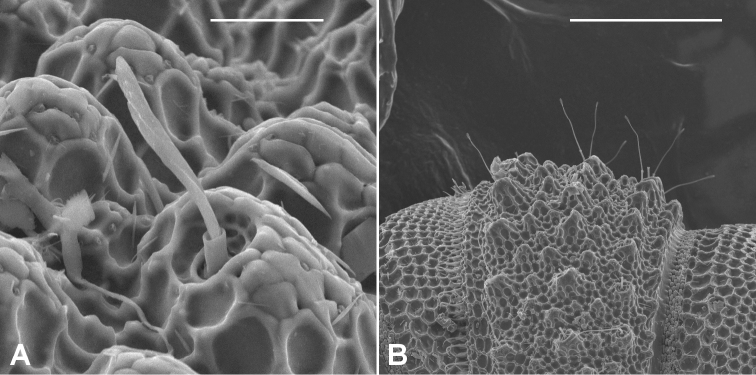
Metatergal setae on midbody rings. **A** *Agathodesmus bucculentus* (Jeekel, 1986), short bipartite seta with slightly flared tip (male ex ANIC 64-000332) **B** *Agathodesmus quintanus* sp. n., very long bipartite setae with slightly flared tips (male paratype ex QM S96066). Scale bars: **A** = 0.02 mm, **B** = 0.2 mm.

Although the metatergal setae in most Australian *Agathodesmus* spp. are short with slightly flared tips (Fig. 4A), three of the newly described species have very long setae (Fig. 4B) and appear 'hairy' at low magnification.

**Figure 5.**
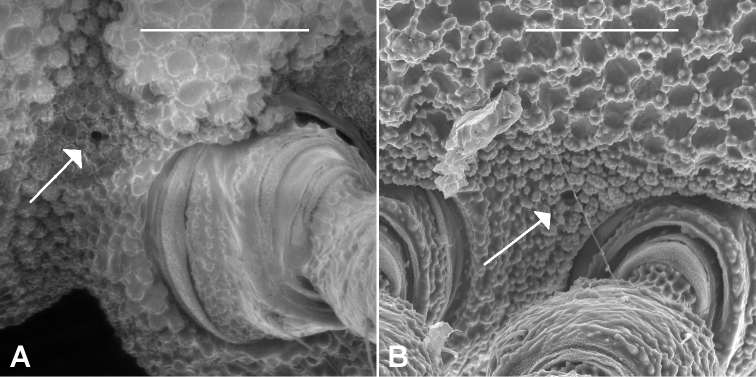
Presumed spiracular openings (arrows) on midbody rings. **A** *Agathodesmus steeli* Silvestri, 1910, AM KS107964, anterior spiracle (anterior to left) **B** *Agathodesmus chandleri* sp. n., posterior spiracle (anterior to right; male paratype ex series SAM OM-2004-2018). Scale bars = 0.25 mm.

While examining the new *Agathodesmus* species I was able to identify what appear to be spiracular openings, something I failed to do earlier (Mesibov 2009). The openings are minute (Fig. 5) and on diplosegments are both located close to the anterior leg base.

Finally, included here as Fig. 10D is a close-up of a typical *Agathodesmus* spp. ozopore, shown at much lower magnification in the *Agathodesmus steeli* redescription (fig. 4B in Mesibov 2009).

#### Agathodesmus adelphus sp. n.

http://zoobank.org/7CDE40B2-0327-40F9-B93D-1679B3824AE4

http://species-id.net/wiki/Agathodesmus_adelphus

Fig. 6A

##### Holotype.

Male, Mt Bartle Frere, Qld, west slopes, 17°22'57"S, 145°46'45"E ±500m, 800-1000 m a.s.l., 30 December 1989, G. Monteith, QM S96015.

##### Paratype.

1 female, details as for holotype, QM S96016.

##### Other material.

None.

##### Diagnostic description.

**Figure 6.**
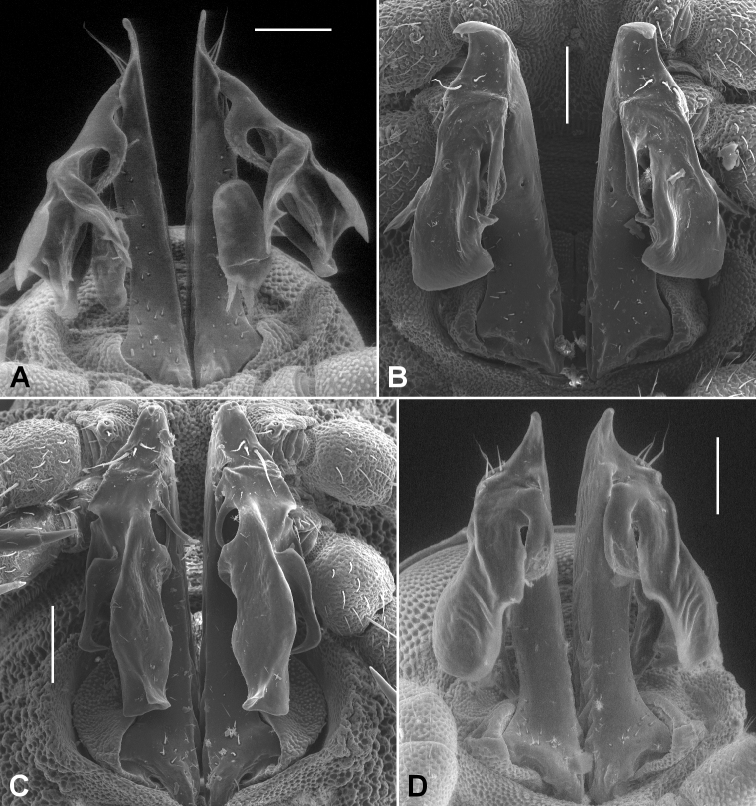
Posterior gonopod views. **A** *Agathodesmus adelphus* sp. n., holotype, QM S96015 **B** *Agathodesmus aenigmaticus* sp. n., paratype ex QM S96018 **C** *Agathodesmus agnus* sp. n., paratype ex QM S96021 **D** *Agathodesmus anici* sp. n., holotype, ANIC 64-000327. **A** and **D** are uncoated specimens; scale bars = 0.1 mm. Note two mites on *Agathodesmus adelphus* sp. n. telopodites.

Male and female with head + 20 rings. Colour in alcohol very faintly reddish. Male/female ca 8.0/8.5 mm long; ring 12 maximum diameter ca 0.6/0.7 mm, maximum width ca 0.85/0.9 mm. Metatergal tubercles in 8-10 irregular transverse rows, mostly without setae; metatergal setae short with slightly flared tips; lateralmost two rows of tubercles not enlarged, together forming narrow pseudo-paranotum with 6 marginal tubercles. Male leg 6 coxa with small, rounded, mediodistal projection. Telopodite (Fig. 6A) with **pp** straight; **at** in longitudinal plane, short and rounded-triangular; **dp** directed laterobasally at base; **mab** concave medially, deeply divided with smaller medial lobe bent posteriorly, tapering and with sharp subterminal tooth on lateral surface; larger, lateral **mab** lobe widening to thickened, emarginate apex; **meb** curving strongly behind **mab**, divided at ca one-third length into 2 thin processes.

##### Distribution.

Known only from rainforest high on Mt Bartle Frere in tropical north Queensland (Fig. 13A). Possibly co-occurs on Mt Bartle Frere with *Agathodesmus quintanus* sp. n. and *Agathodesmus summus* sp. n., although both these species are found at higher elevations on the mountain.

##### Name.

Latin *adelphus*, 'brother', a punning reference to the 'Frere' in the name of the type locality; adjective.

##### Remarks.

Fig. 6A shows two unidentified mites, one on each telopodite. They have not been removed from the holotype.

#### Agathodesmus aenigmaticus sp. n.

http://zoobank.org/E912EEC5-BF65-4E3E-8AA0-024389774BFE

http://species-id.net/wiki/Agathodesmus_aenigmaticus

Fig. 6B

##### Holotype.

Male, Mt Hayward, Qld, 20°19'39"S, 148°45'10"E ±500m, 350 m a.s.l., 20 November 1992, G. Monteith, G. Thompson, D. Cook and H. Janetzki, QM S96017.

##### Paratypes.

8 males, 3 females, 4 stadium 7 males, 2 stadium 7 females, 4 stadium 6 males, 2 stadium 6 females, details as for holotype, QM S96018.

##### Other material.

2 males, Bellenden Ker, Centre Peak, Qld, summit, 17°15'40"S, 145°51'25"E ±500m, 1500 m a.s.l., 11 April 1979, G. Monteith, QM berlesate 17, rainforest, sieved litter, QM S96019.

##### Diagnostic description.

Male and female with head + 20 rings. Colour in alcohol pale yellow. Male/female ca 9.0/10 mm long; ring 12 maximum diameter ca 0.75/0.9 mm, maximum width ca 0.9/1.0 mm. Metatergal tubercles in 8-10 irregular transverse rows, larger tubercles mostly with setae; metatergal setae long, bluntly pointed; 5 lateralmost tubercles slightly enlarged, forming very narrow pseudo-paranotum. Male leg 6 coxa with small, rounded, mediodistal projection. Telopodite (Fig. 6B) with **pp** straight; **at** in transverse plane, short, broad and with rounded apex curving posteriorly; **dp** directed posterobasally at base; **mab** slightly expanded apically with rounded distal margin, lateral edge with small, tapering, posterolaterally directed lobe, medial edge with short, curved lobe partly protecting **meb**; **meb** divided near base into 3 thin, subequal processes curving behind **mab**.

##### Distribution.

Known from rainforest at two localities ca 450 km apart in north Queensland (Figs 12, 13A; see Remarks).

##### Name.

Latin *aenigmaticus*, 'puzzling'; adjective (see Remarks).

##### Remarks.

The very wide disjunction between the two known localities is puzzling, and I suspect that the Bellenden Ker locality on the specimen label is incorrect. I have not noticed any differences in gonopod or non-gonopod features between the two populations.

#### Agathodesmus agnus sp. n.

http://zoobank.org/33CC8023-FD1A-47D3-8A78-F7590C27F670

http://species-id.net/wiki/Agathodesmus_agnus

Figs 6C , 10D

##### Holotype.

Male, Lamb Range, 19 km S of Mareeba, Qld, 17°06'39"S, 145°34'04"E ±500 m, 1200 m a.s.l., 3 December 1988, G. Monteith and G. Thompson, QM berlesate 804, rainforest, sieved litter, QM S96020.

##### Paratypes.

7 males, 10 females, 1 stadium 6 female, details as for holotype, QM S96021.

##### Other material.

**QM:** 1 male, 3 females, 3 stadium 6 females, North Bell Peak via Gordonvale, Qld, 17°05'19"S, 145°52'44"E ±500 m, 900 m a.s.l., 16 September 1981, G. Monteith and D. Cook, QM berlesate 300, rainforest, sieved litter and moss, S96022; 1 male, 1 female, 22 km SE of Mareeba, Qld, 17°06'39"S, 145°34'04"E ±500 m, 900 m a.s.l., 4 November 1983, D. Yeates and G. Thompson, QM berlesate 615, rainforest, sieved litter, S96023; 1 female, 1 stadium 6 female, North Bell Peak, Qld, 17°05'06"S, 145°52'00"E ±500 m, 600 m a.s.l., 22 November 1990, G. Monteith and G. Thompson, QM berlesate 845, rainforest, sieved litter, S96024; 2 males, 2 females, Mt Haig,Lamb Range, Qld, 17°05'52"S, 145°36'09"E ±500 m, 1000 m a.s.l., 25 February 1997, G. Monteith, QM berlesate 918, rainforest, sieved litter, S37557. **ANIC:** 1 male, near Mt Haig, Qld, 17°10'S, 145°36'E ±1 km, 750 m a.s.l., 30 June 1971, R.W. Taylor and J. Feehan, ANIC berlesate 350, rainforest, 64-000323; 2 males, Mt Haig, Qld, 17°06'S, 145°36'E ±1 km, 1000 m a.s.l., 29 October 1976, R.W. Taylor and T.A. Weir, ANIC berlesate 541, rainforest, 64-000325; 15 males, 9 females, 3 km W by S of Mt Haig, Qld, 17°06'S, 145°34'E ±1 km, 1150 m a.s.l., 3 April 1984, A. Calder and T.A. Weir, ANIC berlesate 952, rainforest, 64-000324; 7 males, 2 females, Cammoo Caves near Rockhampton (? – see Remarks), Qld, 23°10'S, 150°28"E ±1 km, 25 October 1976, R.W. Taylor and T.A. Weir, ANIC berlesate 535, dense low closed forest, ANIC 64-000326.

##### Diagnostic description.

Male and female with head + 19 rings. Colour in alcohol pale yellow. Male/female ca 8.0/8.0 mm long; ring 12 maximum diameter ca 0.65/0.8 mm, maximum width ca 0.7/1.1 mm. Metatergal tubercles in ca 12 irregular transverse rows, mostly without setae; metatergal setae short with slightly flared tips; lateralmost tubercles not enlarged, not forming pseudo-paranotum. Male legs 6 coxa with rounded, mediodistal projection. Telopodite (Fig. 6C) with **pp** straight; **at** in transverse plane, short, triangular and curving posteriorly; **dp** directed posterobasally at base; **mab** deeply divided into large posterior and small posterolateral lobes; posterior lobe of **mab** distally with small folds and with medial edge producednear **mab** base as short, pointed lobe; posterolateral lobe of **mab** folded, the posteriormost fold with apex tooth-like and pointing anteromedially; **meb** divided at base into 2 needle-like processes curving behind **mab**.

##### Distribution.

Rainforest in the Lamb Range and adjacent hills inland from Gordonvale, tropical north Queensland (Fig. 13A). Co-occurs with *Agathodesmus quintanus* sp. n. on North Bell Peak. There is also a doubtful record from Cammoo Caves in central Queensland (Fig. 1; see Remarks).

##### Name.

Latin *agnus*, 'lamb', for the type locality, the Lamb Range; adjective.

##### Remarks.

Like *Agathodesmus agnus* sp. n., *Asphalidesmus magnus* Mesibov, 2011 and *Prosopodesmus crater* Mesibov, 2012 were found in ANIC berlesate 535 from Cammoo Caves (Mesibov 2011, 2012a). For all three species, all other specimens are from localities on or near the Lamb Range, ca 800 km to the north of Cammoo Caves. The locality labelling for ANIC berlesate 535 appears to be incorrect (Mesibov 2012a). In March 2013 I searched briefly for millipedes in rainforest in the Cammoo Caves area but found no specimens of *Agathodesmus*, *Asphalidesmus* or *Prosopodesmus*.

#### Agathodesmus anici sp. n.

http://zoobank.org/917051E9-2E87-4FB1-830A-4C5B775DAE1E

http://species-id.net/wiki/Agathodesmus_anici

Fig. 6D

##### Holotype.

Male, 11 km W by N of Bald Hill, McIlwraith Range, Qld, search party campsite, 13°44'S, 143°20"E ±2 km, 520 m a.s.l., 27 June–12 July 1989, T.A. Weir, ANIC berlesate 1111, closed forest, leaf and log litter, in several pieces in genitalia vial, ANIC 64-000327.

##### Other material.

None.

##### Diagnostic description.

Male with head + 20 rings. Colour in alcohol pale white. Male ca 7.5 mm long; ring 12 maximum diameter ca 0.6 mm, maximum width ca 0.75 mm. Metatergal tubercles in 7-8 irregular transverse rows, mainly without setae; metatergal setae long, pointed; 4 lateralmost tubercles not enlarged, forming very narrow pseudo-paranotum. Male leg 6 coxa with prominent mediodistal projection. Telopodite (Fig. 6D) with **pp** straight; **at** in transverse plane, short, narrowly triangular and with tip curving posteriorly; **dp** directed posterobasally and laterally at base; **mab** shallowly divided into narrower anterior and wider posterior lobes; **meb** curving behind **mab** and divided at ca 1/3 length into short, needle-like medial and broader lateral processes, the latter following the curve of the posterior **mab** lobe and nearly as long.

##### Distribution.

Known only from the type locality on the Cape York Peninsula in far north Queensland (Fig. 1).

##### Name.

In honour of ANIC, the Australian National Insect Collection, whose collection of berlesates has yielded many new species of Australian millipedes.

##### Remarks.

The telson of the holotype is damaged and the distal portions of legs 6 are missing.

#### Agathodesmus bonang sp. n.

http://zoobank.org/8687C72E-AFF7-4518-AA89-D51D6D03BB10

http://species-id.net/wiki/Agathodesmus_bonang

Fig. 7A

##### Holotype.

Male, Bonang Road, Vic, 37°23'26"S, 148°35'49"E ±25 m, 320 m a.s.l., 9 April 2011, R. Mesibov, NMV K-11860.

##### Paratypes.

**NMV:** 9 males (K-11861-11869), 5 females (K-11872-11876), 1 stadium 6 female (K-11871), 1 stadium 5 female (K-11870), details as for holotype; 1 stadium 6 female, same details but 37°15'31"S, 148°44'02"E ±25 m, 620 m a.s.l., K-11878; 1 female, same locality but 37°26'01"S, 148°35'47"E ±25 m, 240 m a.s.l., 8 November 2006, R. Mesibov and T. Moule, K-11877.

##### Other material.

None.

##### Diagnostic description.

**Figure 7.**
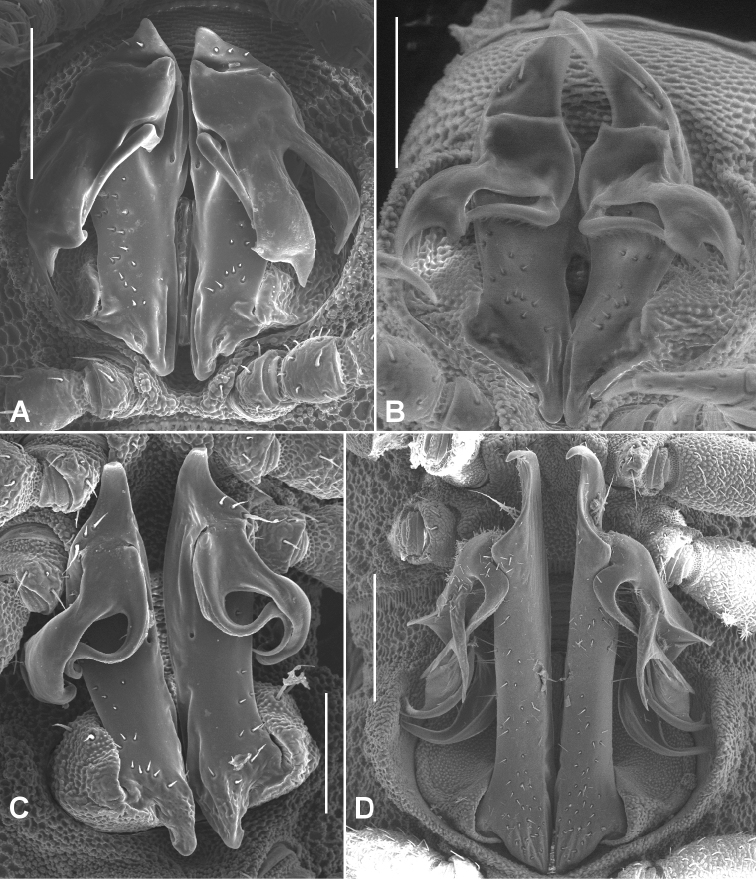
Posterior gonopod views. **A** *Agathodesmus bonang* sp. n., paratype ex series NMV K-11861-11869 **B** *Agathodesmus carorum* sp. n., NMV K-11884 **C** *Agathodesmus chandleri* sp. n., paratype ex series SAM OM-2004-2018 **D** *Agathodesmus hahnensis* sp. n., paratype ex QM S38962. **B** is uncoated specimen; scale bars: **A**, **B**, **C** = 0.1 mm, **D** = 0.2 mm.

Male and female with head + 19 rings. Colour in alcohol pale white. Male/female ca 3.5/4.0 mm long; ring 12 maximum diameter ca 0.3/0.4 mm, maximum width ca 0.4/0.5 mm. Metatergal tubercles in ca 4-5 irregular transverse rows, mostly without setae; metatergal setae short with slightly flared tips; 3 lateralmost tubercles enlarged, forming narrow pseudo-paranotum. Male leg 6 without coxal projection. Telopodite (Fig. 7A) with **pp** straight; **at** in transverse plane, very short, rounded-triangular and bent posteriorly; **dp** directed posterobasally and slightly laterally at base; **mab** deeply and widely divided into 2 subequal lobes with bluntly pointed apices; **meb** not divided, bent first posteriorly, then laterobasally and only very slightly curved, apex behind medial edge of medial lobe of **mab**.

##### Distribution.

Known from wet eucalypt forest in East Gippsland, Victoria, over a linear extent of ca 20 km (Fig. 11).

##### Name.

For the Bonang Highway, a narrow and winding road through the eastern Victorian mountains, type locality of this species; noun in apposition.

##### Remarks.

Like *Agathodesmus carorum* sp. n., *Agathodesmus bonang* sp. n. is white in colour when alive, and contrasted well with the wet leaf litter and rotting wood in which I found it.

#### Agathodesmus carorum sp. n.

http://zoobank.org/7438443E-DBEF-445A-8E3E-6997A72163A0

http://species-id.net/wiki/Agathodesmus_carorum

Fig. 7B

##### Holotype.

Male, Imlay Road, NSW, 37°07'40"S, 149°27'28"E ±25 m, 610 m a.s.l., 11 November 2006, C. Car, G. Car, R. Mesibov and T. Moule, NMV K-11879.

##### Paratypes.

**NMV:** 1 male (K-11882), 1 stadium 6 female (K-11883), Nadgee State Forest, S of Eden, NSW, 37°23'56"S, 149°49'18"E ±25 m, 260 m a.s.l., 12 November 2006, C. Car, G. Car, R. Mesibov and T. Moule; 2 females, same details but 37°24'54"S, 149°48'57"E, 230 m a.s.l., K-11880-11881.

##### Other material.

**NMV:** 1 male (K-11884), 2 females (K-11885), Dyer Creek, near Murrungowar, Vic, 37°38'26"S, 148°43'24"E ±25 m, 170 m a.s.l., 8 November 2006, R. Mesibov and T. Moule; 1 male, Cabbage Palms Flora Reserve, Vic, 37°44'39"S, 148°38'55"E ±25 m, 30 m a.s.l., 10 April 2011, R. Mesibov, K-11886.

##### Diagnostic description.

Male and female with head + 19 rings. Colour in alcohol pale white. Male/female ca 4.0/4.5 mm long; ring 12 maximum diameter ca 0.4/0.5 mm, maximum width ca 0.5/0.6 mm. Metatergal tubercles in ca 6 irregular transverse rows, mostly without setae; metatergal setae short; 3 lateralmost tubercles enlarged, forming narrow pseudo-paranotum. Male leg 6 without coxal projection. Telopodite (Fig. 7B) with **pp** slightly curved posteriorly, widening distally; **at** in transverse plane, long, tapering to blunt point and curving posteromedially; **dp** directed posterobasally at base; **mab** directed posterolaterally at ca 90° to **pp** axis, then curving basally, the distal margin emarginate in its medial portion; **meb** not divided, bent sharply and directed posterolaterally near base, apex behind medial edge of **mab**.

##### Distribution.

Wet eucalypt forest in far southeastern New South and far eastern Victoria (Fig. 11).

##### Name.

In honour of Catherine and George Car, co-collectors of this species at the holotype and paratype localities; adjective.

##### Remarks.

Like *Agathodesmus bonang* sp. n., *Agathodesmus carorum* sp. n. is white in colour when alive.

#### Agathodesmus chandleri sp. n.

http://zoobank.org/F1B036CA-96BE-46DA-B0A6-8EFBC9372B3C

http://species-id.net/wiki/Agathodesmus_chandleri

Figs 5B , 7C

##### Holotype.

Male, Wotton Scrub, Kenneth Stirling Conservation Park, SA, 34°58'58"S, 138°46'39"E ±100 m, 450 m a.s.l., 1 July 2010, R. Mesibov and T. Moule, SAM OM2003.

##### Paratypes.

**SAM:** 15 males, OM2004-OM2018; 5 females, OM2019-OM2023; 2 stadium 6 males, OM2024, OM2025; details as for holotype.

##### Other material.

**SAM:** 2 males, Richardsons Road, Uraidla, SA, 34°58'13"S, 138°45'02"E ±25 m, 580 m a.s.l., 21 August 2010, R. Mesibov and T. Moule, OM2026, OM2027; 1 male, Whites Scrub, SA, 34°58'06"S, 138°46'42"E ±25 m, 510 m a.s.l., same date and collectors, OM2028. **ANIC:** 4 males, 1 female, Pill Box Track, Cleland Conservation Park, 16 km SE of Adelaide, SA, 34°58'S, 138°42'E ±1 km, 500 m a.s.l., 25 April 1993, D.S. Chandler, ANIC berlesate 1539, cut dry sclerophyll, Eucalyptus and grass litter, 64-000337.

##### Diagnostic description.

Males and females with head + 19 rings. Colour in alcohol pale yellow. Male/female ca 5.0/5.5 mm long; ring 12 maximum diameter ca 0.45/0.5 mm, maximum width ca 0.55/0.7 mm. Metatergal tubercles in 6-7 irregular transverse rows, mostly without setae; metatergal setae short with slightly flared tips; 5 lateralmost tubercles enlarged, forming very narrow pseudo-paranotum. Male leg 6 without coxal projection. Telopodite (Fig. 7C) with **pp** straight; **at** in transverse plane, short, narrowly triangular with rounded tip curving posteriorly; **dp** directed posterobasally at base; **mab** directed laterobasally and curving anterobasally, narrow and not divided into lobes, distal margin thickened and with small emargination; **meb** curving behind **mab**, then following anterobasal curve of **mab** and terminating with it.

##### Distribution.

Wet and dry eucalypt forest in the Adelaide Hills east of Adelaide, South Australia (Fig. 1).

##### Name.

In honour of the American entomologist Don Chandler, who collected the first known specimens of this species while on a field trip to Australia; adjective.

##### Remarks.

At the type locality I found an isolated aggregation of individuals of this species in very wet, friable material inside a rotting eucalypt log (http://www.polydesmida.info/polydesmida/thanks.html). Other specimens were in wet litter close to rotting wood.

As with *Agathodesmus* spp. localities elsewhere in eastern Australia, the four *Agathodesmus chandleri* sp. n. localities in the Adelaide Hills all have mean annual rainfalls of at least 1000 mm. This species is likely to be a wet-forest relict surviving on a high-rainfall 'island' in an otherwise dry region of the Australian continent. Nevertheless, *Agathodesmus chandleri* sp. n. appears to be locally abundant in the Adelaide Hills, and occurs in habitats much disturbed by burning and (formerly) stock grazing.

#### Agathodesmus gayundah sp. n.

http://zoobank.org/5390D7BE-197E-46ED-8B07-774D5EFFFD52

http://species-id.net/wiki/Agathodesmus_gayundah

**Figure 8.**
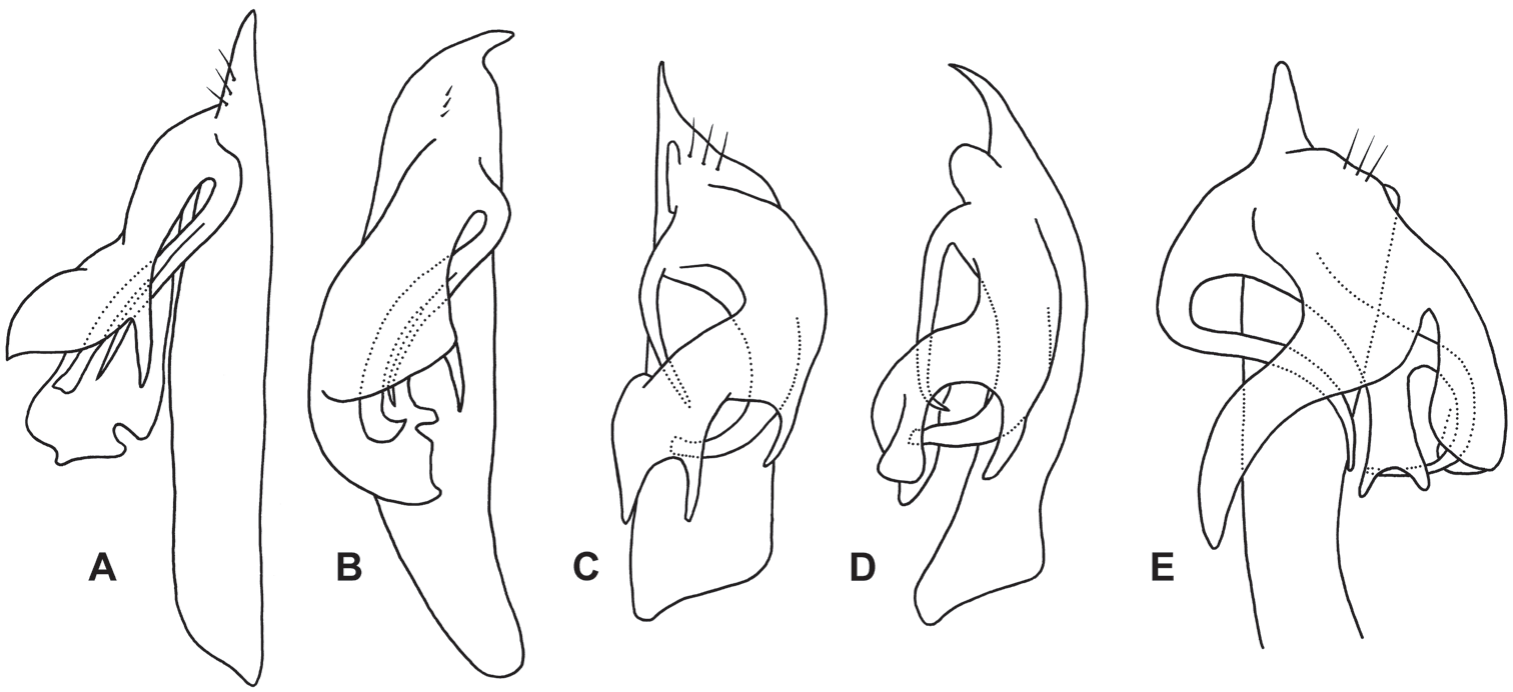
*Agathodesmus* spp. gonopod telopodites, not to same scale. **A**, **B** *Agathodesmus gayundah* sp. n., paratype ex QM S96039, right gonopod, posterior (**A**) and lateral (**B**) views **C**, **D** *Agathodesmus millaa* sp. n., paratype ex QM S96055, left gonopod, posterior (**C**) and lateral (**D**) views, **as** not shown in lateral view **E** *Agathodesmus quintanus* sp. n., paratype ex QM S96074, left gonopod, posterior and slightly basal view.

Figs 3C, 3D , 8A, 8B

##### Holotype.

Male, Gayundah Creek, Hinchinbrook Island, Qld, 18°21'59"S, 146°13'09"E ±500 m, 10 m a.s.l., 11 November 1984, V. Davies, G. Thompson and J. Gallon, QM berlesate 664, rainforest sieved litter, QM S96029.

##### Paratypes.

**QM:** 4 males, 2 females, details as for holotype, S96035; 1 male, 2 females, 3 stadium 7 males, 1 stadium 7 female, 3 stadium 6 males, 4 stadium 6 females, 1 stadium 5 male, 1 stadium 4 male, same details but 9 November 1984, QM berlesate 663, S96033; 1 male, same details but 10 November 1984, QM berlesate 666, S96031; 2 males, 1 female, same details but QM berlesate 668, S96034; 1 male, 1 female, same details but 8 November 1984, G. Monteith, V. Davies, G. Thompson and J. Gallon, QM berlesate 667, S96032; 1 male, same details but QM berlesate 665, S96030; 3 males, same locality, 7-14 November 1984, V. Davies and J. Gallon, S96038; 3 males, 3 females, 2 stadium 7 females, 1 stadium 6 male, same locality, 7–15 November 1984, G. Monteith, G. Thompson and D. Cook, S96039; 3 males, 4 females, 2 stadium 7 males, 4 stadium 7 females, 1 stadium 6 male, 1 stadium 6 female, same locality but 18°21'36"S, 146°13'33"E ±500 m, 80 m a.s.l., 12 November 1984, G. Monteith, V. Davies, G. Thompson and J. Gallon, QM berlesate 669, S96036; 1 female, 2 stadium 7 females, same details but G. Monteith and G. Thompson, S96037.

##### Other material.

None.

##### Diagnostic description.

Male and female with head + 20 rings. Colour in alcohol very pale yellow. Male/female ca 10.5/10.5 mm long; ring 12 maximum diameter ca 0.9/1.1 mm, maximum width ca 1.25/1.3 mm. Metatergal tubercles in 10–12 irregular transverse rows, mainly without setae; metatergal setae short with slightly flared tips; lateralmost row of tubercles enlarged, together with more medial 1-2 rows forming wide pseudo-paranotum with 5-6 marginal tubercles (Figs 3C, 3D). Male leg 6 coxa with small, rounded, mediodistal projection. Telopodite (Figs 8A, 8B) with **pp** slightly flattened mediolaterally, slightly curved posteriorly; **at** in oblique plane (facing posterolaterally), short, narrowly triangular, curving posteriorly; **dp** directed laterobasally at base; **mab** directed basally and a little anteriorly, widening distally and divided into 2 lobes; longer anterior **mab** lobe medially concave with interior folds; shorter posterior **mab** lobe concave medially with flat, spike-like, basally directed process at medial edge; **meb** curving behind **mab**, divided at about one-third length into 2 parallel processes, the shorter posteromedial process needle-like, the longer, wider anterolateral process terminating in triangular tooth.

##### Distribution.

Known only from rainforest on Hinchinbrook Island, east of Cardwell in tropical north Queensland (Fig. 13A).

##### Name.

For the type locality, Gayundah Creek; noun in apposition.

#### Agathodesmus hahnensis sp. n.

http://zoobank.org/DA20F497-5F24-402A-AB92-ED2C01361DF3

http://species-id.net/wiki/Agathodesmus_hahnensis

Fig. 7D

##### Holotype.

Male, Hahn Tableland, N end, Qld, 16°48'35"S, 145°10'53"E ±500 m, 950–1000 m a.s.l., 11-14 December 1995, G. Monteith, G. Thompson and D. Cook, rainforest, QM S96040 (ex S38962).

##### Paratypes.

9 males, 6 females, details as for holotype, QM S38962.

##### Other material.

**QM:** 1 male, Mt Finnigan, 37 km S of Cooktown, Qld, 15°48'53"S, 145°16'28"E ±500 m, 850–1100 m a.s.l., 22 April 1982, G. Monteith, D. Yeates and D. Cook, rainforest, S96044; 1 male, Mossman Bluff track, 9 km W of Mossman, Qld, 16°27'52"S, 145°17'12"E ±500 m, 1000 m a.s.l., 22-24 December 1989, G. Monteith and G. Thompson, pitfall trap, S96042; 1 male, 1 stadium 7 female, 10 km N of Mt Lewis, Qld, 16°29'29"S, 145°15'10"E ±500 m, 1100 m a.s.l., 25 November 1990, G. Monteith, G. Thompson, D. Cook, R. Sheridan and H. Janetzki, S96043; 1 male, 1 female, Mt Misery road, Qld, 15°52'39"S, 145°12'58"E ±500 m, 730 m a.s.l., 2 January 1991, ANZSES personnel, S37591; 1 female, Graham Range, Qld, 17°16'24"S, 145°57'58"E ±500 m, 550 m a.s.l., 1 November 1995, G. Monteith, pyrethrum, trees and logs, S96041; 2 males, same details but 8-9 December 1995, G. Monteith, G. Thompson and D. Cook, S38960. **ANIC:** 1 male, Windsor Tableland, 1.2 km past barracks, Qld, 16°15'10"S, 145°02'30"E ±500 m, 1060 m a.s.l., 8 February 1998, G. Monteith and D. Cook, ANIC berlesate 1831, 64-000338.

##### Diagnostic description.

Male and female with head + 20 rings. Colour in alcohol very pale yellow. Male/female ca 9.0/10.0 mm long; ring 12 maximum diameter ca 0.8/1.0 mm, maximum width ca 0.9/1.2 mm. Metatergal tubercles in 10-12 irregular transverse rows, mainly without setae; metatergal setae short with slightly flared tips; lateralmost row of tubercles enlarged, together with more medial 1-2 rows forming prominent pseudo-paranotum with 6 marginal tubercles. Male leg 6 coxa with small, rounded, mediodistal projection. Telopodite (Fig. 7D) with **pp** straight, slightly flattened mediolaterally; **at** in oblique plane (facing posterolaterally), short, narrowly triangular, curving posteriorly; **dp** directed laterobasally at base; **mab** deeply divided into 3 lobes: 2 anterolateral lobes curving anteriorly, then posterolaterally, narrowing and with upturned apices, and a basally directed posteromedial lobe widely divaricate at mid-length; **meb** curving behind **mab**, divided at ca one-third length into 2 closely appressed, needle-like processes, terminating between divaricate posteromedial and curved anterolateral lobes of **mab**.

##### Distribution.

Rainforest in tropical north Queensland from Mt Finnigan near Cooktown south to the Graham Range, a linear extent of ca 180 km (Fig. 13A). Co-occurs with *Agathodesmus quintanus* sp. n. in the Graham Range.

##### Name.

For the type locality, the Hahn Plateau; adjective.

#### Agathodesmus kerensis sp. n.

http://zoobank.org/92DA65F0-C022-4642-99AD-BE9835F8EE72

http://species-id.net/wiki/Agathodesmus_kerensis

Fig. 9A

##### Holotype.

Male, Bellenden Ker Range, Qld, summit TV station, 17°15'50"S, 145°51'14"E ±100m, 1550 m a.s.l., 25-31 October 1981, Queensland Museum and Earthwatch personnel, QM S96045.

##### Paratypes.

**QM:** 2 males, 1 stadium 6 female, details as for holotype, S96046; 3 males, same details but 1–7 November 1981, S96047; 2 males, same details but 1–7 November 1981, QM berlesate 338, rainforest, sieved litter, S96048.

##### Other material.

None.

##### Diagnostic description.

**Figure 9.**
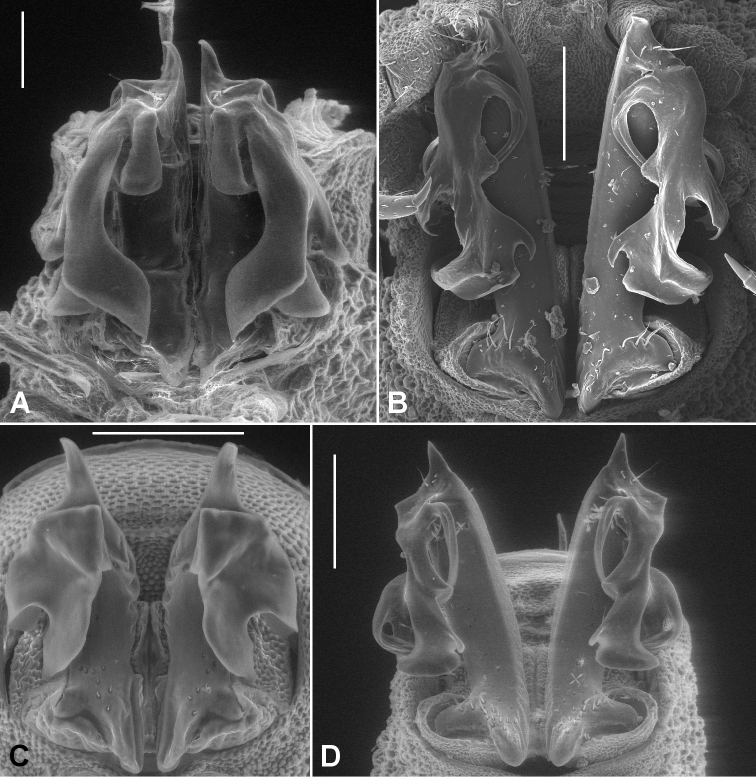
Posterior gonopod views. **A** *Agathodesmus kerensis* sp. n., paratype ex QM S96046 **B** *Agathodesmus kirrama* sp. n., paratype ex QM S96051 **C** *Agathodesmus morwellensis* sp. n., paratype, NMV K-11889 **D** *Agathodesmus parapholeus* sp. n., paratype ex QM S96057. **A**, **C**, **D** are uncoated specimens; scale bars: **A**, **C** = 0.1 mm, **B**, **D** = 0.2 mm.

Male with head + 19 rings. Colour in alcohol brownish yellow. Male ca 8.0 mm long; ring 12 maximum diameter ca 0.6 mm, maximum width ca 0.65 mm. Metatergal tubercles in ca 10 irregular transverse rows, mainly without setae; metatergal setae short with slightly flared tips; lateralmost row of tubercles not enlarged, not forming pseudo-paranotum. Male leg 6 coxa with small, rounded, mediodistal projection. Telopodite (Fig. 9A) with **pp** curving anteriorly, then distally at base, slightly flattened anteroposteriorly; **at** in oblique plane (facing posterolaterally), short, narrowly triangular, arising abruptly from medial side of truncate **pp** apex, curving slightly posterolaterally; **dp** directed posterobasally at base; **mab** divided into 2 distally notched lobes, the posterior lobe curving medially and terminating in a basally directed point; **meb** broad, not divided, curving behind **mab**, apex slightly expanded.

**Figure 10.**
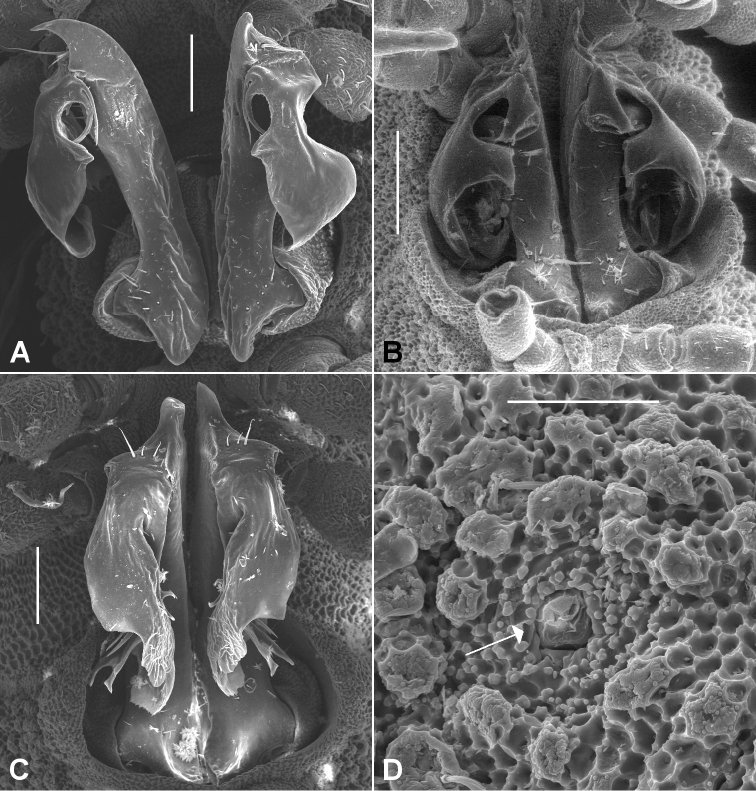
**A**–**C**, posterior gonopod views. **A** *Agathodesmus sagma* sp. n., paratype ex QM S96076 **B** *Agathodesmus summus* sp. n., paratype ex QM S96091 **C** *Agathodesmus yuccabinensis* sp. n., paratype ex QM S96094 **D** Midbody ozopore (arrow) of *Agathodesmus agnus* sp. n., male paratype ex QM S96021. **B** is uncoated specimen; scale bars: **A**, **C** = 0.1 mm, **B** = 0.2 mm, **D** = 0.05 mm.

**Figure 11.**
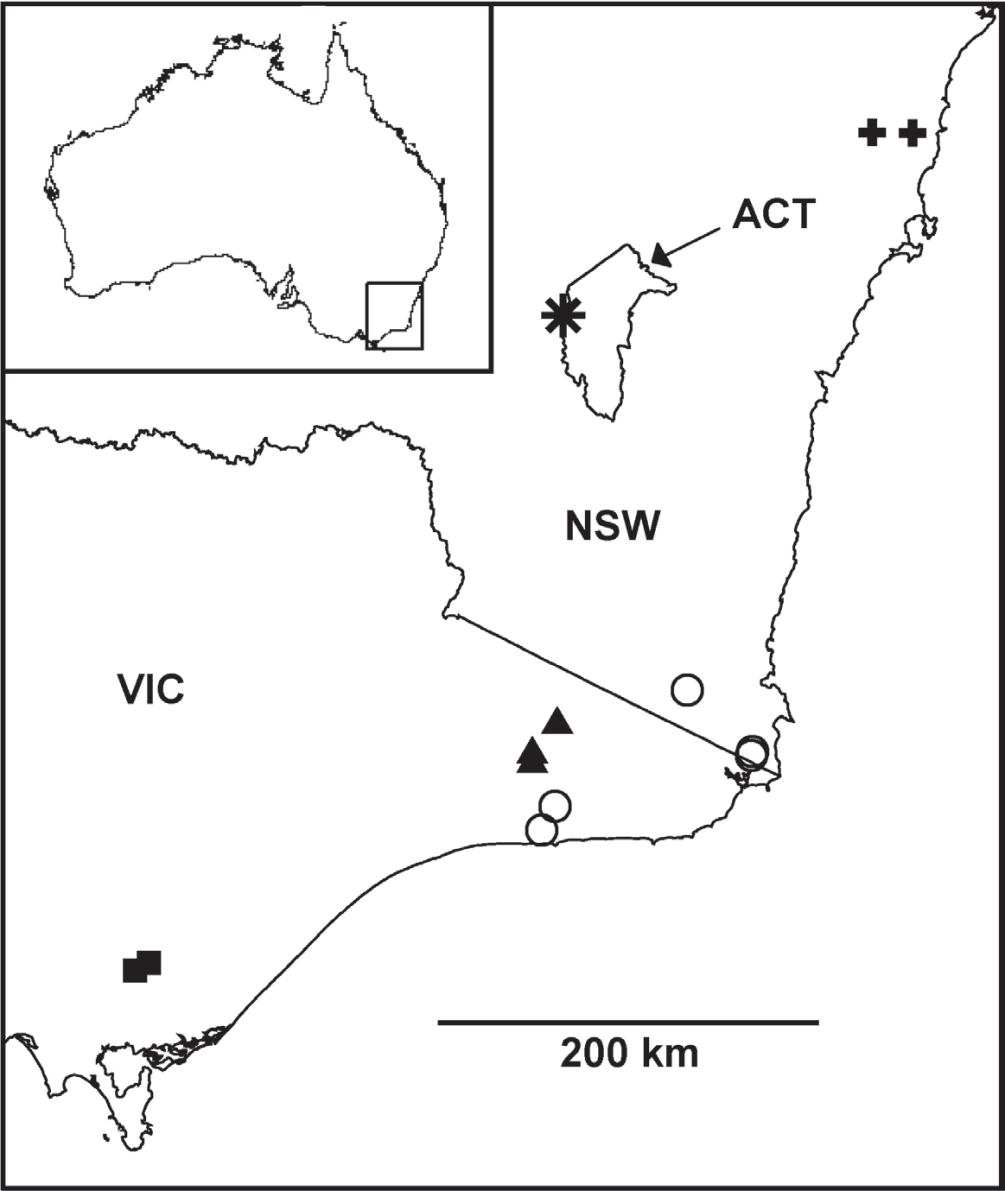
Localities for *Agathodesmus bonang* sp. n. (filled triangles), *Agathodesmus carorum* sp. n. (open circles), *Agathodesmus johnsi* Mesibov, 2009 (star), *Agathodesmus morwellensis* sp. n. (filled squares) and *Agathodesmus steeli* Silvestri, 1910 (crosses). Mercator projection; ACT = Australian Capital Territory, NSW = New South Wales, VIC = Victoria. Inset shows location of main map.

**Figure 12.**
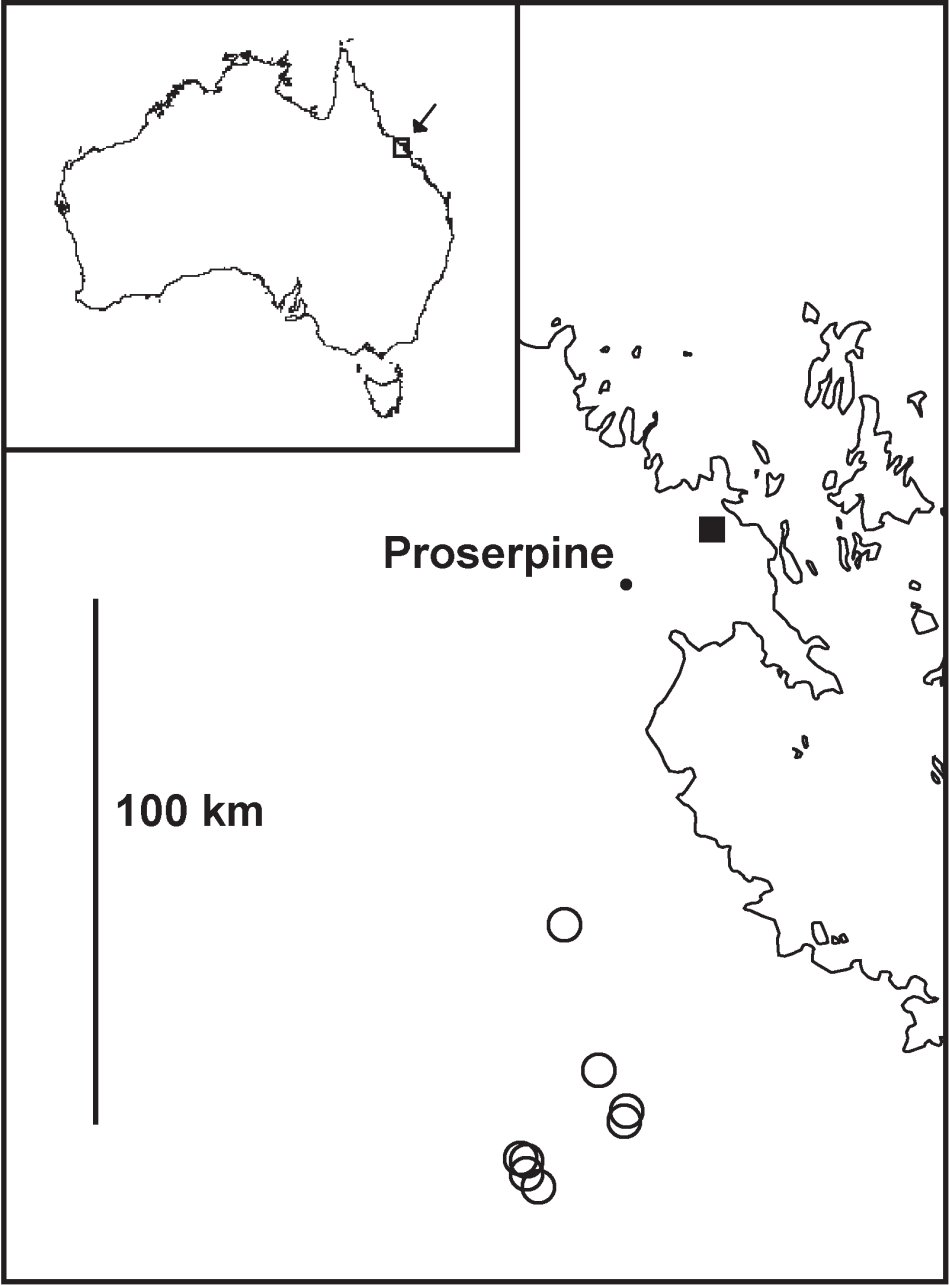
Localities for *Agathodesmus aenigmaticus* sp. n. (filled square) and *Agathodesmus bucculentus* (Jeekel, 1986) (open circles) in central coastal Queensland. Mercator projection; inset shows location of main map.

**Figure 13.**
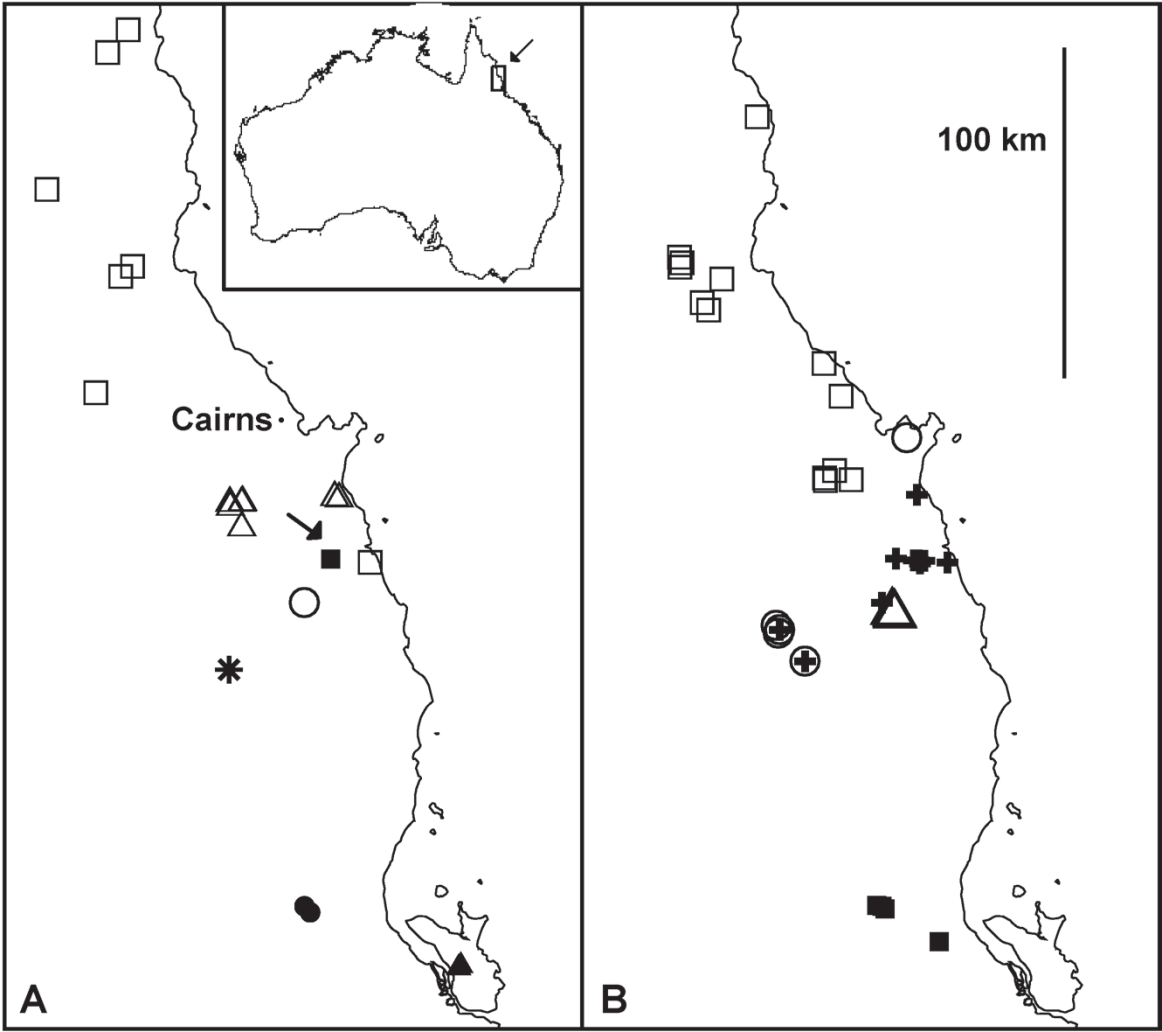
Localities in far north Queensland for **A** *Agathodesmus adelphus* sp. n. (open circle), *Agathodesmus agnus* sp. n. (open triangles), *Agathodesmus gayundah* sp. n. (filled triangle), *Agathodesmus hahnensis* sp. n. (open squares), *Agathodesmus kerensis* sp. n. (filled square), *Agathodesmus kirrama* sp. n. (filled circles), *Agathodesmus millaa* sp. n. (star) and **B** *Agathodesmus parapholeus* sp. n. (open circles), *Agathodesmus quintanus* sp. n. (crosses), *Agathodesmus sagma* sp. n. (open squares), *Agathodesmus summus* sp. n. (open triangles), and *Agathodesmus yuccabinensis* sp. n. (filled squares). Arrow in **A** indicates questionable, disjunct locality for *Agathodesmus aenigmaticus* sp. n. Mercator projections; inset shows location of main maps.

##### Distribution.

Known only from rainforest on the summit of Mt Bellenden Ker in tropical north Queensland (Fig. 13A). *Agathodesmus quintanus* sp. n. occurs lower down on the same mountain.

##### Name.

For the type locality, Mt Bellenden Ker; adjective.

##### Remarks.

Latitude/longitude data corrected following Mesibov (2012b).

#### Agathodesmus kirrama sp. n.

http://zoobank.org/2D19A715-D908-40DD-80C4-4DA9EAC3A65F

http://species-id.net/wiki/Agathodesmus_kirrama

Fig. 9B

##### Holotype.

Male, Mt Pershouse, Kirrama Range, Qld, 18°13'30"S, 145°47'42"E ±500m, 12 December 1986, G. Monteith and G. Thompson, QM berlesate 734, rainforest, sieved litter, QM S96049.

##### Paratypes.

**QM**: 4 males, 1 female, 1 stadium 6 male, details as for holotype, S96051; 2 males, 2 females, same details but QM berlesate 735, S96050.

##### Other material.

**QM**: 1 male, 2 females, Mt Hosie, Kirrama Range Qld, 18°12'29"S, 145°46'41"E ±500m, 800 m a.s.l., 10 December 1986, G. Monteith and G. Thompson, S96052; 2 males, same details but 930 m a.s.l., 11 December 1986, QM berlesate 733, rainforest, sieved litter, S96053.

##### Diagnostic description.

Male and female with head + 19 rings. Colour in alcohol pale yellow. Male/female ca 10.0/10.5 mm long; ring 12 maximum diameter ca 0.75/0.8 mm, maximum width ca 0.8/0.9 mm. Metatergal tubercles in 12-16 irregular transverse rows, mainly without setae; metatergal setae short with slightly flared tips; lateralmost row of tubercles not enlarged, not forming pseudo-paranotum. Legs 6 and 7 coxae with long mediodistal projections (shorter on leg 7) with rounded tips. Telopodite (Fig. 9B) with **pp** straight; **at** in transverse plane, short and narrowly triangular, tip not curving posteriorly (curve in Fig. 9B is artefact of drying); **dp** directed posterobasally and laterally at base; **mab** divided into 3 lobes increasing in width and length from anterior to posterior, with anterior lobe curving medially and pointed, middle lobe distally truncate, posterior lobe distally expanded, the distal margin curving cup-like anteriorly and with small notch near medial edge; **meb** curving behind **mab**, divided at ca one-quarter length into 2 subequal, closely appressed, needle-like processes.

##### Distribution.

Known only from rainforest on Mt Hosie and Mt Pershouse, ca 3 km apart in tropical north Queensland (Fig. 13A). Co-occurs with *Agathodesmus yuccabinensis* sp. n. on Mt Hosie.

##### Name.

For the Kirrama Range; noun in apposition.

#### Agathodesmus millaa sp. n.

http://zoobank.org/6D607416-62E9-4A33-8687-1A390D172A19

http://species-id.net/wiki/Agathodesmus_millaa

Figs 8C, 8D

##### Holotype.

Male, Mt Fisher, 7 km SW of Millaa Millaa, Qld, Whiteing Road, 17°33'56"S, 145°33'47"E ±500m, 1200 m a.s.l., 5 May 1983, G. Monteith and D. Yeates, QM berlesate 583, moss on rocks and logs; in 3 pieces in genitalia vial, QM S96054.

##### Paratypes.

1 male, 1 stadium 5 male, details as for holotype, QM S96055.

##### Other material.

None.

##### Diagnostic description.

Male with head + 19 rings. Colour in alcohol pale yellow. Male ca 7.5 mm long; ring 12 maximum diameter ca 0.5 mm, maximum width ca 0.6 mm. Metatergal tubercles in ca 10-12 irregular transverse rows, mainly without setae; metatergal setae short with slightly flared tips; lateralmost row of tubercles not enlarged, not forming pseudo-paranotum. Male leg 6 without coxal projection. Telopodite (Figs 8C, 8D) with **pp** curving posteriorly, anteroposteriorly flattened (wider in posterior view than lateral); **at** in oblique plane (facing posterolaterally), short, narrowly triangular, curving posterolaterally; **dp** directed posterobasally and laterally at base, a small, rounded, mediolaterally flattened tab arising on posterior surface just above (basal to) **mab** origin; **mab** divided into 2 lobes; lateral **mab** lobe terminating in basomedially curving, finger-like process; medial **mab** lobe curving medially, divided into broad medial and narrow, distally expanded lateral process with truncate distal margin; **meb** curving behind **mab**, divided near base into needle-like, basally directed medial process and long, broad lateral process curving medially and terminating in short, broad hook behind medial lobe of **mab**.

##### Distribution.

Known only from rainforest at the type locality on the Atherton Tableland in tropical north Queensland (Fig. 13A).

##### Name.

For the type locality, Millaa Millaa; noun in apposition.

#### Agathodesmus morwellensis sp. n.

http://zoobank.org/71AF8186-F2FF-483F-AA02-F76DA644A211

http://species-id.net/wiki/Agathodesmus_morwellensis

Fig. 9C

##### Holotype.

Male, Fosters Gully, Morwell National Park, Vic, 38°21'41"S, 146°23'20"E ±25 m, 170 m a.s.l., 31 July 2006, R. Mesibov, in pieces in genitalia vial, NMV K-11887.

##### Paratypes.

**NMV:** 1 female, details as for holotype, K-11888; 1 male (K-11889), 1 female (K-11890), E of Churchill, Vic, 38°19'39"S, 146°27'59"E ±25 m, 190 m a.s.l., same date and collector.

##### Other material.

None.

##### Diagnostic description.

Male and female with head + 19 rings. Colour in alcohol pale white. Male/female ca 4.5/5.0 mm long; ring 12 maximum diameter ca 0.4/0.5 mm, maximum width ca 0.5/0.6 mm. Metatergal tubercles in ca 5 irregular transverse rows, mainly without setae; metatergal setae very short; 4 lateralmost tubercles enlarged, forming narrow pseudo-paranotum. Male leg 6 without coxal projection. Telopodite (Fig. 9C) with **pp** gently curving posteriorly, somewhat fusiform; **at** in transverse plane, short, narrowly triangular, curving posteriorly; **dp** directed posterobasally at base; **mab** folded outwards at base, shallowly divided into 2 lobes; **meb** short, broad, pointed, bent behind **mab**, not visible in posterior view.

##### Distribution.

Known from eucalypt forest in the Latrobe River valley in West Gippsland, Victoria, at two sites ca 7 km apart (Fig. 11). Non-male specimens (in NMV) only tentatively assigned to *Agathodesmus* have been found in the mountains northwest of the type locality.

##### Name.

For the type locality, Morwell National Park; adjective.

#### Agathodesmus parapholeus sp. n.

http://zoobank.org/F6965A14-A93B-4EB9-9F09-B201AF32BAFA

http://species-id.net/wiki/Agathodesmus_parapholeus

Fig. 9D

##### Holotype.

Male, Tower near The Crater, Qld, 17°27'23"S, 145°29'12"E ±500 m, 1230 m a.s.l., 23 November 1994, G. Monteith, QM berlesate 878, rainforest, sieved litter, QM S96056 (ex QM S46994).

##### Paratypes.

**QM:** 12 males, 4 females, 1 stadium 6 male, 1 stadium 6 female, details as for holotype, S96057; 1 male, 2 females, same details but 16 May 1995, QM berlesate 886, rainforest, stick brushing, S96058.

##### Other material.

**QM:** 1 female, 21 km S of Atherton, Qld, 17°26'41"S, 145°28'35"E ±500 m, 1000 m a.s.l., 5 November 1983, D. Yeates and G. Thompson, QM berlesate 616, rainforest, sieved litter, S96059; 3 males, 1 female, same details but QM berlesate 617, S96060; 1 male, 1 female, Kjellberg Road, Mt Fisher, Qld, 17°32'34"S, 145°33'31"E ±500 m, 1100 m a.s.l., 18 May 1995, G. Monteith, QM berlesate 892, rainforest, sieved litter, S96061; 16 males, 5 females, 2 stadium 6 females, 2 stadium 5 females, Mt Murray Prior, Qld, 16°55'59"S, 145°50'59"E ±500 m, 770 m a.s.l., 30 October 1995, G. Monteith, QM berlesate 894, rainforest, sieved litter, QM S96062. **ANIC:** Longlands Gap Road, 27 km S of Atherton, Qld, 17°28'S, 145°29'E ±2 km, 11 November 1969, J.G. Brooks, ANIC berlesate 169, leaf mould, rainforest, 64-000339.

##### Diagnostic description.

Male and female with head + 19 rings. Colour in alcohol pale yellow. Male/female ca 8.0/9.0 mm long; ring 12 maximum diameter ca 0.75/0.85 mm, maximum width ca 0.75/0.85 mm. Metatergal tubercles in ca 12 irregular transverse rows, mainly without setae; metatergal setae short with slightly flared tips; lateralmost tubercles not enlarged, not forming pseudo-paranotum. Male leg 6 coxa with prominent mediodistal projection. Telopodite (Fig. 9D) with **pp** straight; **at** in transverse plane, short, rounded-triangular, tip not curving posteriorly; **dp** directed posterobasally and laterally at base; **mab** deeply divided into 2 lobes; anterior **mab** lobe subdivided, its posterior portion curving medially; posterior **mab** lobe with cup-like partial fold at midlength, the distal margin bent into narrow shelf and with small notch near medial edge; **meb** curving behind **mab**, divided at base into 2 subequal, closely appressed, needle-like processes.

##### Distribution.

Known from rainforest on the Atherton Tableland and the coastal range just southeast of Cairns in tropical north Queensland (Fig. 13B); the two areas are ca 70 km apart. Co-occurs with *Agathodesmus quintanus* sp. n. on the Atherton Tableland.

##### Name.

Greek *para*, 'near', and *pholeos*, 'hole'; adjective. The type locality is close to Mt Hypipamee Crater, a vertical volcanic pipe.

##### Remarks.

*Agathodesmus parapholeus* sp. n. is very similar to *Agathodesmus kirrama* sp. n. (compare Figs 9B and 9D). I am separating them on the number of **mab** lobes (three in *kirrama* sp. n. vs two in *parapholeus* sp. n.) and the development of long mediodistal projections on the leg 6 coxae in *kirrama* sp. n. These differences may be clinal, and further collecting would be useful in the ca 70 km-wide gap between closest known occurrences of the two species.

#### Agathodesmus quintanus sp. n.

http://zoobank.org/71796952-F691-4A75-B90F-B564EA88E414

http://species-id.net/wiki/Agathodesmus_quintanus

Figs 4B , 8E

##### Holotype.

Male, Bellenden Ker Range, Qld, cable tower 5, 17°16'04"S, 145°53'00"E ±100 m, 500 m a.s.l., 17-24 October 1981, Queensland Museum and Earthwatch personnel, QM S96063.

##### Paratypes.

**QM:** 4 males, 4 females, details as for holotype, S96064; 2 males, same locality but cableway base station, 17°16'06"S, 145°53'54"E ±100 m, 110 m a.s.l., 25-31 October 1981, same collectors, S96067; 2 males, 1 female, same details but QM berlesate 309, rainforest, stick brushings, S96073; 1 male, 1 female, 1 stadium 6 female, same locality but 1 km S of cable tower 6, 17°16'33"S, 145°53'15"E ±100 m, 500 m a.s.l., 17-24 October 1981, same collectors, QM berlesate 319, rainforest, sieved litter, S96074; 1 male, 1 female, 1 stadium 6 male, 1 stadium 6 female, 1 stadium 5 male, same details but 25-31 October 1981, QM berlesate 321, rainforest, sieved litter, S96068; 3 males, same locality but cable tower 3, 17°16'02"S, 145°52'12"E ±100 m, 1020 m a.s.l., 17-24 October 1981, same collectors, S96066.

##### Other material.

**QM:** 1 male, 2 females, 1 stadium 6 female, North Bell Peak via Gordonvale, Qld, 17°05'19"S, 145°52'44"E ±500 m, 900 m a.s.l., 16 September 1981, G. Monteith and D. Cook, QM berlesate 300, rainforest, sieved litter and moss, QM S96065; 1 male, Massey Range, Qld, 17°15'45"S, 145°49'06"E ±500 m, 1250 m a.s.l., 10 October 1991, G. Monteith and H. Janetzki, QM berlesate 853, rainforest, sieved litter, S96069; 1 male, 2 females, Tower near The Crater, Qld, 17°27'23"S, 145°29'12"E ±500 m, 1230 m a.s.l., 23 November 1994, G. Monteith, QM berlesate 878, rainforest, sieved litter, ex S46994, S96070; 2 males, 1 female, Kjellberg Road, Mt Fisher, Qld, 17°32'34"S, 145°33'31"E ±500 m, 1100 m a.s.l., 18 May 1995, G. Monteith, QM berlesate 892, rainforest, sieved litter, S96071; 2 males, Graham Range, Qld, 17°16'24"S, 145°57'58"E ±500 m, 550 m a.s.l., 1 November 1995, G. Monteith, QM berlesate 895, rainforest, sieved litter, S96072. **ANIC:** 1 male, Bartle Frere Track, 17 km E of Malanda, Qld 17°22'57"S, 145°46'45"E ±500 m, 1200 m a.s.l., 8 December 1988, G. Monteith and G. Thompson, QM berlesate 815, rainforest, sieved litter, 64-000340 [handwritten ANIC label incorrectly gives '17 km W' and '1989']

##### Diagnostic description.

Male and female with head + 19 rings. Colour in alcohol very pale yellow. Male/female ca 5.5/6.0 mm long; ring 12 maximum diameter ca 0.6/0.65 mm, maximum width ca 0.8/0.85 mm. Metatergal tubercles in 4-5 irregular transverse rows, larger tubercles mainly with setae; metatergal setae long with slightly flared tips (Fig. 4B); 3 lateralmost tubercles enlarged, forming narrow pseudo-paranotum. Male leg 6 coxa with small, rounded, mediodistal projection. Telopodite (Fig. 8E) with **pp** curving gently posteriorly; **at** in transverse plane, short, rounded-triangular, curving posteriorly; **dp** directed posterobasally at base; **mab** greatly widening distally, divided into 2 lobes; lateral **mab** lobe directed anterobasally, distally spoon-shaped, concave medially; medial **mab** lobe produced basomedially with roundly pointed tip; **meb** large and complex, divided into 2 lobes; medial **meb** lobe curving widely mediobasally at base, then laterobasally, then basally, tapering to blunt point, the curve at base often overlapping the initial curve of the contralateral medial **meb** lobe; lateral **meb** lobe directed basolaterally, divided at about midlength into flat, basally directed, distally deeply emarginate tab, and long, needle-like process curving first posteriorly, then anterobasally, terminating anterior to **pp**.

##### Distribution.

Rainforest southwest of Babinda in tropical north Queensland, with a known east-west extent of ca 50 km (Fig. 13B). Co-occurs with *Agathodesmus hahnensis* sp. n. in the Graham Range and with *Agathodesmus parapholeus* sp. n. near Mt Hypipamee Crater. Possibly co-occurs on Mt Bartle Frere with *Agathodesmus adelphus* sp. n.

##### Name.

Latin *quintanus*, 'of the fifth'; adjective. The type locality was recorded as the fifth tower supporting the cableway to the top of Mt Bellenden Ker. This tower has since been renumbered '4' (Mesibov 2012b)

##### Remarks.

*Agathodesmus quintanus* sp. n. has a particularly complicated gonopod telopodite and I am not certain that I have clearly seen all its details. The species is distinguished by the wide initial curve of the medial **meb** lobe and the basomedially directed, roundly pointed medial **mab** lobe.

Specimens from North Bell Peak are larger than those from the type locality and have more prominent dorsal tubercles. Latitude/longitude data for the types have been corrected following Mesibov (2012b).

#### Agathodesmus sagma sp. n.

http://zoobank.org/6C431D39-25DB-4C23-BF3D-97860D5C331C

http://species-id.net/wiki/Agathodesmus_sagma

Fig. 10A

##### Holotype.

Male, Saddle Mountain, Qld, summit, 16°49'11"S, 145°39'42"E ±500 m, 650 m a.s.l., 21 November 1994, G. Monteith, QM berlesate 877, rainforest, sieved litter, QM S96075.

##### Paratypes.

6 males, 1 female, 1 stadium 6 female, details as for holotype, QM S96076.

##### Other material.

**QM:** 1 male, Mt Demi, 7.0 km S of Mossman, Qld, 16°29'54"S, 145°19'13"E ±500 m, 900–1000 m a.s.l., 26 April 1983, G. Monteith and D. Yeates, QM berlesate 546, rainforest, litter, S96085; 1 male, 2.5 km N of Mt Lewis via Julatten, Qld, 16°33'49"S, 145°15'51"E ±500 m, 1040 m a.s.l., 3 November 1983, D. Yeates and G. Thompson, QM berlesate 611, rainforest, sieved litter, S96077; 1 male, 1 female, 1 stadium 5 male, 2 km SE of Mt Spurgeon via Mt Carbine, Qld, 16°27'17"S, 145°12'26"E ±500 m, 1100 m a.s.l., 20 December 1988, G. Monteith and G. Thompson, QM berlesate 825, rainforest, sieved litter, S96078; 1 male, Lambs Head, 10 km W of Edmonton, Qld, campsite, 17°01'23"S, 145°38'33"E ±500 m, 1200 m a.s.l., 12 December 1989, G. Monteith, G. Thompson and H. Janetzki, QM berlesate 835, rainforest, litter, S96086; 1 male, 1 female, same details but 10 December 1989 to 8 January 1990, rainforest pitfalls, S96087; 4 males, 1 female, Davies Creek Road, Qld, 17°02'33"S, 145°36'51"E ±500 m, 750 m a.s.l., 17 December 1989, G. Monteith and G. Thompson, QM berlesate 836, rainforest, sieved litter, S96079; 2 males, 2 females, same details but QM berlesate 841, S96080; 1 male, 6 females, 1 stadium 6 male, Mt Formartine South, Qld, 16°43'48"S, 145°36'45"E ±500 m, 700 m a.s.l., 24 November 1990, G. Monteith and G. Thompson, QM berlesate 848, rainforest, sieved litter, S96081; 1 male, same details but 23–24 November 1990, pitfall traps, S96084; 6 males, 4 females, Mt Halcyon, Qld, 16°03'16"S, 145°25'16"E ±500 m, 870 m a.s.l., 24 November 1993, G. Monteith and H. Janetzki, QM berlesate 864, rainforest, sieved litter, S96082; 5 males, 1 female, Isley Hills, Qld, 17°02'52"S, 145°41'25"E ±500 m, 1050 m a.s.l., 1 December 1993, G. Monteith and H. Janetzki, QM berlesate 866, rainforest, sieved litter and moss, S96083; 1 male, 1 female, 2 stadium 6 males, Mt Spurgeon, Qld, summit, 16°26'22"S, 145°12'00"E ±500 m, 1300 m a.s.l., 22 November 1997, G. Monteith, QM berlesate 956, rainforest, sieved litter, S46128; 1 male, 3 km S of Mt Spurgeon, Qld, 16°27'59"S, 145°12'02"E ±500 m, 1140 m a.s.l., 19-23 November 1997, G. Monteith, D. Cook and C. Burwell, sclerophyll forest, S35882. **ANIC:** 1 male, Mt Lewis, Qld, 16°35'S, 145°17'E ±1 km, 960 m a.s.l., 30 October 1976, R.W. Taylor and T.A. Weir, ANIC berlesate 545, rainforest, 64-000341; 5 males, 2 km N by E of Mt Tiptree, Qld, 17°03'S, 145°37'E ±1 km, 1 April 1984, A. Calder and T.A. Weir, ANIC berlesate 950, rainforest, 64-000343; 4 males, Mt Tiptree, Qld, 17°03'S, 145°37'E ±1 km, 13 July 1984, B. Halliday, ANIC berlesate 1006, rainforest, leaf litter, 64-000342.

##### Diagnostic description.

Male and female with head + 19 rings. Colour in alcohol very pale yellow. Male/female ca 8.0/8.0 mm long; ring 12 maximum diameter ca 0.7/0.8 mm, maximum width ca 0.7/0.85 mm. Metatergal tubercles in 10-12 irregular transverse rows, mainly without setae; metatergal setae short with slightly flared tips; lateralmost tubercles not enlarged, not forming pseudo-paranotum. Male leg 6 coxa with small, rounded, mediodistal projection. Telopodite (Fig. 11A) with **pp** straight; **at** in oblique plane (facing posterolaterally), short, rounded-triangular, curving posterolaterally; **dp** directed posterobasally and laterally at base; **mab** somewhat expanded distally, divided into 2 lobes with large anterior fold; **meb** divided at base into 2 needle-like processes, the smaller medial process directed basally, the longer medial process curving behind **mab**.

##### Distribution.

Wet forest in tropical north Queensland from the Cape tribulation area south to the Atherton Tableland, a north-south extent of ca 120 km (Fig. 13B).

##### Name.

Latin *sagma*, 'saddle'; noun in apposition. For the type locality, Saddle Mountain.

#### Agathodesmus summus sp. n.

http://zoobank.org/0FFE6498-73F7-4CBD-9C82-24473D6FF6A7

http://species-id.net/wiki/Agathodesmus_summus

Fig. 10B

##### Holotype.

Male, Mt Bartle Frere, Qld, centre peak ridge, 17°23'27"S, 145°48'33"E ±500 m, 1400–1500 m a.s.l., 7-8 November 1981, Queensland Museum and Earthwatch personnel, QM berlesate 358, rainforest, sieved litter, QM S96088.

##### Paratypes.

**QM:** 2 males, 2 females, 1 stadium 7 female, details as for holotype, S96091; 2 males, same locality and collectors but S peak summit, 17°24'03"S, 145°49'00"E ±500 m, 1620 m a.s.l., 6-8 November 1981, QM berlesate 359, rainforest, sieved litter, S96090; 1 female, same details but QM berlesate 354, S96089; 2 females, same locality but top camp, 17°23'47"S, 145°48'53"E ±500 m, 1500 m a.s.l., 29 November 1998, G. Monteith, pyrethrum knockdown, S96092.

##### Other material.

None.

##### Diagnostic description.

Male with head + 19 rings, female with head + 20. Colour in alcohol very pale yellow. Male/female ca 9.0/9.5 mm long; ring 12 maximum diameter ca 0.75/0.9 mm, maximum width ca 0.8/1.0 mm. Metatergal tubercles in 10–12 irregular transverse rows, mainly without setae; metatergal setae short with slightly flared tips; lateralmost tubercles not enlarged, not forming pseudo-paranotum. Male leg 6 without coxal projection. Telopodite (Fig. 11B) with **pp** slightly curving posteriorly; **at** in oblique plane (facing posterolaterally), short, narrowly triangular, curving posterolaterally; **dp** directed laterobasally and slightly posteriorly at base; **mab** expanded distally, divided into 2 distally rounded lobes, the medial lobe with truncate medial projection at ca midlength; **meb** curving behind **mab** and nearly as long, divided at ca midlength into shorter, needle-like lateral process and broad medial process terminating in upturned hook.

##### Distribution.

Known only from rainforest at the type locality in tropical north Queensland (Fig. 13B). Possibly co-occurs on Mt Bartle Frere with *Agathodesmus adelphus* sp. n. and *Agathodesmus quintanus* sp. n.

##### Name.

Latin *summus*, 'highest'; adjective. This species was found at the top of Queensland's highest mountain, Mt Bartle Frere.

##### Remarks.

The Bartle Frere females were assigned to this species rather than to the co-occurring *Agathodesmus adelphus* sp. n. and *Agathodesmus quintanus* sp. n. because the females lack the narrow pseudo-paranota found in the other two species.

#### Agathodesmus yuccabinensis sp. n.

http://zoobank.org/024140B1-5299-4539-9873-898BD7CC8D55

http://species-id.net/wiki/Agathodesmus_yuccabinensis

Fig. 10C

##### Holotype.

Male, near Yuccabine Creek, Kirrama Range, Qld, 18°12'21"S, 145°45'47"E ±500 m, 700 m a.s.l., 10 December 1986, G. Monteith and G. Thompson, QM berlesate 732, rainforest, sieved litter, QM S96093.

##### Paratypes.

3 males, 7 females, 1 stadium 6 female, details as for holotype, QM S96094.

##### Other material.

**QM:** 2 males, 1 female, Kirrama Range, Qld, 18°12'57"S, 145°47'15"E ±500 m, 700 m a.s.l., G. Monteith and G. Thompson, QM berlesate 730, rainforest, sieved litter, S96095; 3 males, Mt Hosie, Kirrama Range, Qld, 18°12'29"S, 145°46'41"E ±500 m, 930 m a.s.l., 11 December 1986, same collectors and method but QM berlesate 733, S96096; 3 males, 2 females, Mt Macalister, Cardwell Range, Qld, 18°18'16"S, 145°56'32"E ±500 m, 1000 m a.s.l., 20 December 1986, same collectors and method but QM berlesate 739, S96098; 1 male, 1 stadium 6 male, 1 stadium 6 female, same details but 700 m a.s.l., QM berlesate 741, moss on trees and rocks, S96097; 1 female, same locality but 900 m a.s.l., 15 January 1987, S. Hamlet, QM berlesate 757, rainforest, sieved litter, S96099.

##### Diagnostic description.

Male and female with head + 19 rings. Colour in alcohol pale yellow. Male/female ca 9.0/9.5 mm long; ring 12 maximum diameter ca 0.7/0.85 mm, maximum width ca 0.8/0.9 mm. Metatergal tubercles in 10-12 irregular transverse rows, mainly without setae; metatergal setae short with slightly flared tips; lateralmost tubercles not enlarged, not forming pseudo-paranotum. Male leg 6 without coxal projection. Telopodite (Fig. 11C) with **pp** straight; **at** in transverse plane, short, rounded-triangular, tip curving very slightly posteriorly; **dp** directed posterobasally at base; **mab** curving medially, then basally, the apex divided into truncate lobe with fine marginal teeth, and apically forked lobe; **meb** broad, curving behind **mab** and nearly as long, divided at midlength into short, rounded lateral lobe and 2 slender, subequal, pointed posterior processes reaching almost to **mab** apex.

##### Distribution.

Rainforest in the Cardwell Range (includes Kirrama Range), inland between Tully and Ingham in tropical north Queensland (Fig. 13B). The linear extent of the known range is ca 20 km. Co-occurs with *Agathodesmus kirrama* sp. n. on Mt Hosie.

##### Name.

For Yuccabine Creek, the type locality; adjective.

#### Agathodesmus bucculentus

(Jeekel, 1986)

http://species-id.net/wiki/Agathodesmus_bucculentus

Figs 3A, 3B , 4A

##### Note.

This species was described from three specimens collected by Dr and Mrs Jeekel in 1980, under logs in rainforest along the 'Broken Hill' [probably 'Broken River'] track in Eungella National Park (Jeekel 1986). Another 183 specimens from the same general area are in Australian collections (see below), and the *Agathodesmus bucculentus* range in central coastal Queensland is now known to have a north-south extent of ca 50 km (Fig. 12).

##### Material examined.

**ANIC:** 35 males, 17 females, 1 stadium 7 female, Eungella National Park, Qld, 21°09'S, 148°30'E ±1 km, 760 m a.s.l., 10 November 1976, R.W. Taylor and T.A. Weir, ANIC berlesate 562, rainforest, 64-000332; 16 males, same details but ANIC berlesate 563, 64-000329; 27 males, same details but ANIC berlesate 564, 64-000333; 4 males, Finch Hatton Gorge, Qld, 21°05'S, 148°38'E ±1 km, 200 m a.s.l., 11 November 1976, R.W. same collectors, ANIC berlesate 565, rainforest, 64-000334; 6 males, 4 females, 2 stadium 7 males, 5 stadium 7 females, Broken River, Eungella National Park, Qld, 21°10'S, 148°31'E ±1 km, 700 m a.s.l., 10-12 November 1976, same collectors, ANIC berlesate 559, rainforest, 64-000331; 3 males, 1 female, same details but ANIC berlesate 560, 64-000330; 17 males, 1 female, 1 stadium 7 female, same details but ANIC berlesate 561, 64-000335; 7 males, 1 female, 1 stadium 7 female, same details but ANIC berlesate 568, 64-000328; 18 males, 2 females, same details but ANIC berlesate 570, 64-000336. **QM:** 1 male, 2 females, Mt William, Eungella National Park, Qld, 21°01'05"S, 148°35'57"E ±500 m, 1240 m a.s.l., 19 April 1979, G. Monteith, QM berlesate 41, rainforest, sieved litter, S96025; 2 males, 1 stadium 7 female, Eungella, Qld, schoolhouse, 21°07'51"S, 148°29'32"E ±2 km, 13 February 1986, J. Gallon and R. Raven, QM berlesate 709, rainforest, S96026; 1 male, Finch Hatton Gorge, Qld, 21°04'13"S, 148°38'11"E ±500 m, 300 m a.s.l., 18 November 1992, G. Monteith, G. Thompson, D. Cook and H. Janetzki, S96027; 3 males, 4 females, Mt Macartney, Qld, 20°49'57"S, 148°33'07"E ±500 m, 950 m a.s.l., 19 November 1992, same collectors, S96028.

##### Remarks.

There is little to add to the excellent description and illustrations of Jeekel (1986), except to confirm that the short metatergal setae noted by Jeekel are bipartite with slightly flared tips (Fig. 4A) as in most other *Agathodesmus*, and that the **dp** in *Agathodesmus bucculentus* is indeed 'complicated'. Fig. 16 in Jeekel (1986) clearly shows the **meb**: it is divided near its base into a curving, needle-like medial process and a more or less parallel, broader, distally expanded and lamellar lateral process. However, the **meb** in *Agathodesmus bucculentus* is not 'on caudal side' (Jeekel 1986, p. 49), but lies between the **mab** and the **pp**, as in other *Agathodesmus* spp.

## Supplementary Material

XML Treatment for Agathodesmus

XML Treatment for Agathodesmus adelphus

XML Treatment for Agathodesmus aenigmaticus

XML Treatment for Agathodesmus agnus

XML Treatment for Agathodesmus anici

XML Treatment for Agathodesmus bonang

XML Treatment for Agathodesmus carorum

XML Treatment for Agathodesmus chandleri

XML Treatment for Agathodesmus gayundah

XML Treatment for Agathodesmus hahnensis

XML Treatment for Agathodesmus kerensis

XML Treatment for Agathodesmus kirrama

XML Treatment for Agathodesmus millaa

XML Treatment for Agathodesmus morwellensis

XML Treatment for Agathodesmus parapholeus

XML Treatment for Agathodesmus quintanus

XML Treatment for Agathodesmus sagma

XML Treatment for Agathodesmus summus

XML Treatment for Agathodesmus yuccabinensis

XML Treatment for Agathodesmus bucculentus

## Appendix

Specimen records of Australian *Agathodesmus* species. (doi: 10.3897/zookeys.325.5932.app) File format: Comma Separated Value File (csv).
